# Economic Burden of Post-COVID Syndrome in Germany Between 2021 and 2025: A Statutory Health Insurance Claims Data Analysis

**DOI:** 10.36469/001c.170196

**Published:** 2026-10-21

**Authors:** Daniel Gensorowsky, Emma Fröling, Kylie Braegelmann, Jana Diekmannshemke, Agnes Kisser, Philipp Reuken, Jessica Noll, Julia Blaurock, Eveline Otte im Kampe

**Affiliations:** 1 Vandage GmbH, Bielefeld, Germany; 2 Pfizer Pharma GmbH, Berlin, Germany; 3 Universitätsklinikum Jena, Jena, Germany

**Keywords:** COVID-19, post-COVID, long COVID, healthcare resource utilization, economic burden, cost of illness, sick leave

## Abstract

**Background:**

Evidence on the economic burden of post-COVID syndrome in Germany remains limited. This study quantifies post-COVID-associated healthcare resource utilization (HCRU), sick leave, and costs.

**Objectives:**

To compare HCRU, sick leave, and costs borne by the statutory health insurance (SHI) among incident pediatric and adult post-COVID patients with matched post-COVID-free controls.

**Methods:**

This retrospective cohort study analyzed an anonymized German SHI claims database provided by GWQ ServicePlus AG. The observation period spanned October 2021 to June 2025 and included all individuals with incident COVID-19 diagnosis until September 2024. Incident post-COVID was defined as the first ICD-10-GM code (U07.4!; U09.9!) recorded ≥90 days after first COVID-19 diagnosis. Post-COVID cases were exactly matched 1:2 to post-COVID–free COVID-19 controls by calendar quarter of incident COVID-19 diagnosis, acute severity, cohort entry quarter, 5-year birth interval, sex, and Elixhauser Comorbidity Index. We estimated mean differences in HCRU, sick leave, and direct SHI-borne costs per case. Rate ratios of HCRU, sick leave, and costs per person-year were estimated using Poisson (HCRU/sick leave) and linear (costs) regression. Analyses covered 365-day, 730-day, and any follow-up period.

**Results:**

We matched 25 485 identified post-COVID cases with 49 671 controls. Matched cases and controls were comparable in age, sex, and comorbidities. Overall, during 365 days of follow-up, post-COVID patients showed higher HCRU and sick leave rates across all sectors, especially sick leave days. Mean differences per case were greatest for sick leave days (365 days: +27 days) and outpatient physician contacts (365 days: 6.9 contacts). HCRU and sick leave rates remained significantly increased over extended follow-up periods. Total SHI-borne costs per person-year were higher among post-COVID patients across all follow-up periods (all *P* < .001). Sector-specific costs per person-year were increased primarily in the outpatient sector and for sickness benefits (all *P* < .001). Mean total cost differences per case were +€1806 per case (365 days) and mean excess sickness benefits per case were +€1077.

**Conclusions:**

Documented post-COVID was associated with a substantial and sustained economic burden on the German healthcare system and society. Targeted strategies are needed to mitigate potential long-term impacts on HCRU and labor productivity.

## BACKGROUND

Coronavirus disease 2019 (COVID-19), caused by severe acute respiratory syndrome coronavirus 2 (SARS-CoV-2), has led to considerable morbidity burden extending beyond the acute stage of infection.^1^ Some individuals continue to experience symptoms, or develop new ones, after the initial illness, including cognitive impairment, headache, dyspnea, chest pain, anxiety or depression, myalgia, and joint pain.^2–4^ These sequelae are commonly referred to as long COVID or post-COVID.^5^

However, the terminology and operational definitions for these conditions are not used consistently across the literature and major health authorities.^6^ The German Robert Koch Institute (RKI) and the National Institute for Health and Care Excellence (NICE) use *long COVID* as an umbrella term for health problems or symptoms that persist or develop beyond 4 weeks after the onset of acute COVID-19, whereas *post-COVID condition* or *post-COVID-19 syndrome* refers to symptoms persisting or newly occurring beyond 12 weeks.^7,8^ By contrast, the World Health Organization (WHO) uses the terms *long COVID* and *post COVID-19 condition* largely interchangeably, defining the condition as occurring in individuals with a history of probable or confirmed SARS-CoV-2 infection, usually 3 months from the onset of COVID-19, with symptoms that last for at least 2 months and cannot be explained by an alternative diagnosis.^9^

Although incidence estimates of post-COVID vary significantly based on the definition and study design,^10^ approximately 10% to 15% of COVID-19 patients develop post-COVID.^11,12^ Estimates of the absolute patient numbers are substantial, with estimates as high as 757 000 prevalent cases of post-COVID in Germany at the end of 2025.^13^ Concurrently, estimates of the nationwide economic burden of post-COVID are high, including €136 million of cumulative direct healthcare costs in 2021^14^ and €3.4 billion of production loss in 2021.^15^ Given the high numbers of post-COVID cases and potential for high economic burden, post-COVID presents an ongoing challenge for society, and understanding the drivers of the ongoing economic burden is crucial.

During and in the immediate aftermath of the COVID-19 pandemic, there was a well-documented increase in healthcare resource utilization (HCRU) associated with post-COVID: studies from Germany,^16^ Switzerland,^17^ and England^18^ found that post-COVID and long COVID are associated with higher HCRU across all healthcare sectors. Furthermore, evidence indicates that long-COVID patients have increased HCRU for up to 2 years post-diagnosis.^19^ Commensurate with higher HCRU, estimates of healthcare costs due to post-COVID compared with non-COVID controls range from 1.46 to 1.82 times higher over 6 months in US data^20^ up to twofold in data from the UK.^18^ However, evidence compiled in a 2025 international literature review reported more modest cost increases between 7.6% and 13.1% per post-COVID patient per month when compared with non-COVID-19 controls using differences-in-differences models.^19^

Moreover, in addition to the economic burden in outpatient and inpatient care, post-COVID is associated with a significant, long-term burden in terms of reduced ability to work^21^ and use of sick leave.^22,23^ In a German survey, 41% of working-age individuals being treated for post-COVID reported being unable to work,^24^ although another German study found that only 5.8% of patients used sick leave longer than 4 weeks.^25^ In the literature, risk factors for longer sick leave due to post-COVID include female sex,^25^ older age,^22,23,25^ underlying conditions,^14,22^ and hospitalization during the acute phase,^23^ although evidence from Spain found no difference in sick leave between hospitalized and nonhospitalized long COVID patients.^22^

Recognizing the continued clinical and societal salience of post-COVID, the German Federal Joint Committee (Gemeinsamer Bundesausschuss, G-BA) introduced the Long COVID Guideline (LongCOV-RL), which came into effect on 9 May 2024. This guideline established a framework for coordinated, structured care for individuals with suspected long COVID or post-COVID conditions.^26^ However, inconsistent coding practices, varying presentation of symptoms, and diverse clinical pathways still complicate real-world analyses.

Given the potential for an ongoing burden to healthcare systems and society, this study aims to enhance the understanding of the economic burden associated with diagnosed post-COVID in Germany. We have provided an important longitudinal perspective using current data, reflecting the ongoing burden after the COVID-19 pandemic. Using a nationwide representative anonymized statutory health insurance (SHI) claims database, we estimated HCRU, sick leave, and direct SHI-borne costs associated with diagnosed post-COVID among pediatric and adult individuals from 2021 to 2025.

## METHODS

### Database

The study used SHI claims data from the anonymized data pool provided by GWQ ServicePlus AG. This database comprises routine billing data from 20 small and medium-sized SHI funds operating nationwide in Germany and includes up to 8.4 million observable insured individuals. For each individual, the database contains demographic information as well as comprehensive records on SHI-covered healthcare services (outpatient, inpatient, prescription drugs, prescription medical aids), sick leave, and corresponding direct costs borne by the SHI. These intersectoral person-level claims data provide broad national coverage and are well suited to studying HCRU, sick leave, and SHI-borne costs associated with post-COVID in routine care in Germany.

### Study Design

We conducted a retrospective matched cohort study to estimate HCRU, sick leave, and direct SHI-borne costs associated with post-COVID over a total observation period from 1 October 2020 to 30 June 2025 (**Figure 1**). The period from 1 October 2020 to 30 September 2021 was solely used for washout, exclusion and covariate assessment.

**Figure 1.**
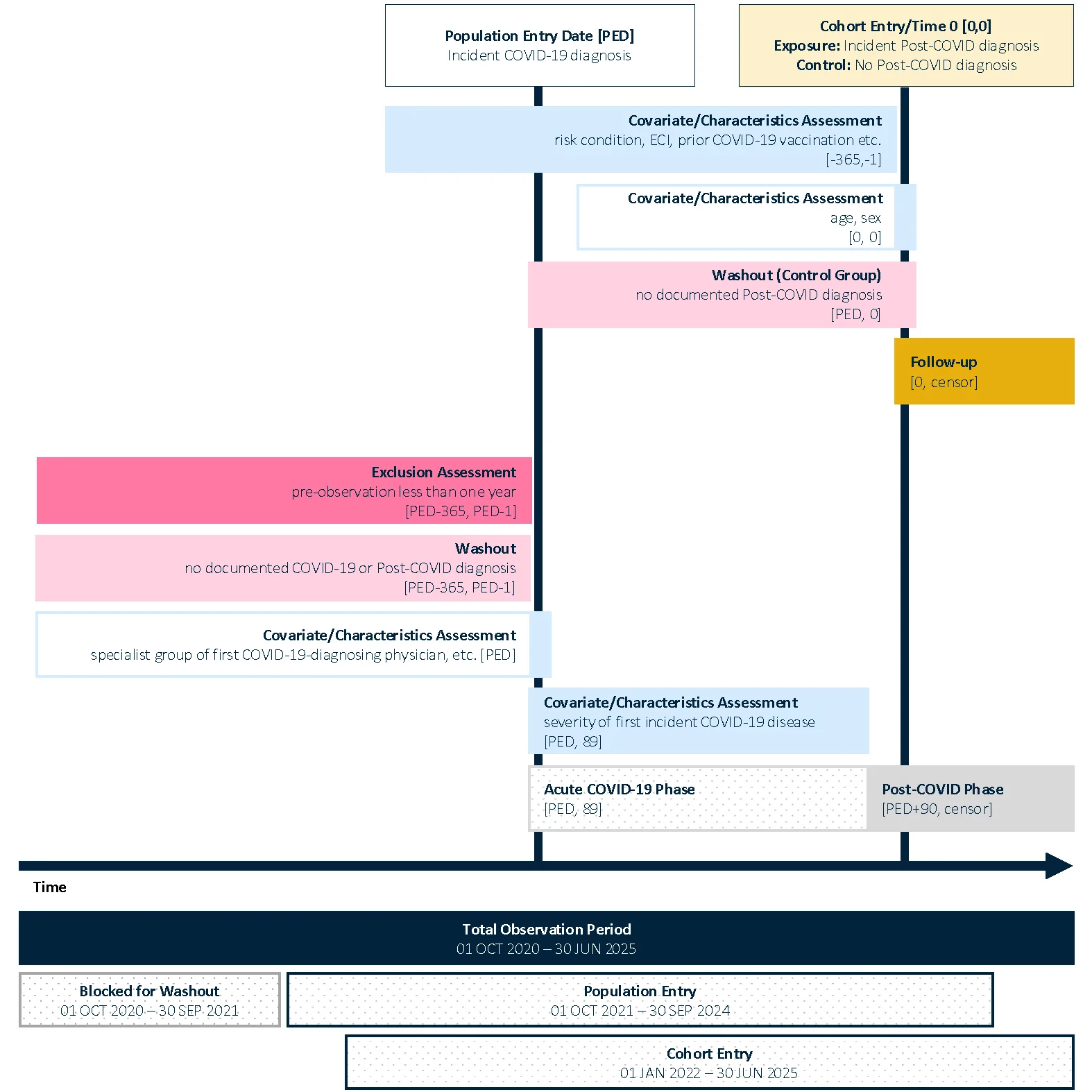
Study Design Diagram for the Analyses of Post-COVID–Associated HCRU and Healthcare Costs

The analyses were nested within a source population of SHI-insured individuals of all ages and sexes with a first documented COVID-19 diagnosis during the COVID-19 seasons 2021/22 and 2023/24 (ie, 1 Oct. 2021–30 Sept. 2024). ICD-10-GM code U07.1 (COVID-19, virus identified) was used to identify acute COVID-19 patients. Other COVID-19–related ICD-10-GM codes that do not indicate laboratory confirmation or do not reflect acute infection were used solely for washout purposes. Incidence was validated by a 365-day washout period prior to the population entry date during which neither COVID-19 nor post-COVID diagnoses may have been documented.

In line with other case definitions, we defined the acute COVID-19 phase as the first 90 days starting with the date of the incident COVID-19 diagnosis.^1,7–9^ The exposed cohort comprised individuals with a first documented post-COVID diagnosis during the subsequent post-acute COVID-19 phase, identified by ICD-10-GM post-COVID codes (before 1 Jan. 2021: U07.4, Post COVID-19 condition; 1 Jan. 2021 onward: U09.9, Post COVID-19 condition, unspecified). Cohort entry for exposed individuals was the date of the first qualifying post-COVID diagnosis during the period from 1 January 2022 to 30 June 2025. Only inpatient diagnoses and confirmed outpatient diagnoses were considered for the COVID-19 population and post-COVID cohort.

The controls comprised individuals with prior incident COVID-19 but no documented post-COVID diagnosis up to the matched case’s cohort entry date. For controls, the same cohort entry date was assigned as the cohort entry date of the matched post-COVID case. Post-COVID cases were exactly matched without replacement (target matching ratio 1:2) to post-COVID–free (post-acute) COVID-19 controls by calendar quarter of incident COVID-19 diagnosis, acute COVID-19 severity, calendar quarter of cohort entry, 5-year birth interval, sex, and Elixhauser Comorbidity Index (ECI). If 2 eligible controls were not available, 1:1 matching was allowed. We reported cohort characteristics before and after matching. Because exact matching was applied, balance on the matching variables was ensured within matched sets by design; standardized mean differences for these variables were therefore not used as the primary matching diagnostic. Matching performance was assessed based on the proportion of cases for which an eligible exact match could be identified and the achieved matching ratio.

Population characteristics were assessed at the COVID-19 population entry date (age, sex, specialty of first COVID-19 diagnosing physician, calendar quarter and season of first COVID-19 diagnosis), during the 365 days before COVID-19 cohort entry (ECI, underlying risk factor for severe COVID-19, COVID-19 vaccination prior to first diagnosed COVID-19), at post-COVID cohort entry date (age, specialty of post-COVID diagnosing physician), during the 365 days before post-COVID cohort entry (ECI, underlying risk factor for severe COVID-19, COVID-19 vaccination prior to first diagnosed COVID-19) and, for severity of the first COVID-19 episode, during the 90-day acute phase of the disease (**Figure 1**).

We analyzed 3 follow-up periods: 365 days, 730 days, and any follow-up through the end of data availability. Follow-up started at post-COVID cohort entry date and ended at the earliest of death, disenrollment from SHI, end of the prespecified follow-up period, or end of observation (30 June 2025). In the control cohort, follow-up also ended at the first documented post-COVID diagnosis. Controls who received a documented post-COVID diagnosis during follow-up were eligible to enter the exposed cohort at the time of that diagnosis and could then be matched to their own controls.

In a sensitivity analysis, we additionally censored controls at the first newly occurring diagnosed post-COVID-associated health outcome to reduce potential misclassification from under-recorded post-COVID diagnoses. These diagnoses had to be absent during the 365-day baseline period before the incident COVID-19 diagnosis and during the acute phase of the disease. Post-COVID–associated health outcomes included domains from the long COVID/post-acute sequelae of COVID-19 patient-reported outcome instrument (Long COVID/PASC PRO) developed by Chandler et al^27^ and additional post-acute COVID-19 events (PACE) such as cardiovascular events, cerebrovascular events, or thromboembolic events (see **Supplementary Material**). Comprehensive code lists provided by Rößler et al^1^ and Yang et al^28^ were used for operationalization through ICD-10-GM codes.

### Study Population

The study included SHI-insured individuals of all ages and sexes who received an incident COVID-19 diagnosis during the COVID-19 seasons 2021/22–2023/24. To ensure a valid assessment of washout and study variables, individuals with an available pre-observation period of less than 365 days prior to the population entry date (ie, date of the first documented COVID-19 diagnosis) were excluded.

### Outcome Measures and Variables

We examined all-cause HCRU, sick leave, and SHI-borne healthcare and sickness benefit costs rather than restricting outcomes to services explicitly coded as post-COVID–related. Analyses were conducted from the perspective of the German SHI. The underlying claims data comprised outpatient and inpatient healthcare claims, outpatient prescription drug and medical aid claims, and information on sick leave, sickness benefits, and the corresponding SHI-borne direct costs.

HCRU was assessed separately by healthcare sector. Outpatient HCRU included the number of outpatient physician contacts and outpatient emergency department (ED) visits. Inpatient HCRU included the number of hospitalizations, distinguished according to whether an intensive care unit (ICU) admission occurred during the hospitalization, and the cumulative number of hospital days. Pharmaceutical and medical aid utilization were measured as the number of outpatient drug and medical aid prescriptions, respectively. Sick leave was analyzed separately from HCRU and was measured as the cumulative number of documented sick leave days during follow-up.

Cost outcomes were assessed from the SHI payer perspective and included direct outpatient, inpatient, prescription drug, medical aid, and sickness benefit costs recorded in the claims data. Costs were assigned to the respective sector based on the underlying type of SHI claim and expressed in euros, with total SHI-borne costs calculated as the sum across sectors (including costs for sickness benefits). No adjustment for inflation was applied; therefore, costs represent nominal euro amounts recorded during the respective observation periods.

Cohort characteristics and covariates included age, sex, ECI, prevalent underlying risk condition for severe COVID-19, COVID-19 vaccination prior to first diagnosed COVID-19, severity of first incident COVID-19, specialist group of the first COVID-19 diagnosing physician and the post-COVID diagnosing physician, season of first COVID-19 diagnosis, length of follow-up, and number of COVID-19 reinfections during follow-up.

Comorbidity burden was summarized using the ECI,^29^ categorized as 0, 1, 2, 3, 4, or ≥5 conditions. Risk conditions were defined as the presence of at least 1 chronic condition associated with an increased risk of severe COVID-19. Conditions were selected based on the definitions provided by RKI.^30^ Codes used for operationalization were based on previous work of Stremel et al,^31^ excluding age, smoking, and pregnancy. Acute COVID-19 severity was categorized as not hospitalized, hospitalized without ICU admission, or hospitalized with ICU admission.

Detailed code lists used for the operationalization of study variables are provided in the **Supplementary Material**. Operational definitions of study variables are summarized in **Supplementary Table A**.

### Statistical Analysis

For descriptive analyses, we summarized HCRU, sick leave days and costs as mean values with standard deviations (SD) and mean differences between groups. Because follow-up could be censored by death or disenrollment, we estimated mean cumulative HCRU, sick leave, and costs using a discrete-time version of the Zhao and Tian estimator based on monthly intervals.^32^ Descriptive results were stratified by length of follow-up (365 days and 730 days), age group, prevalent underlying risk condition for severe COVID-19, and severity of acute COVID-19.

For inferential analyses, we estimated associations between post-COVID and each HCRU outcome/sick leave days using Poisson regression models and report exponentiated coefficients as rate ratios with 95% confidence intervals (CI). The logarithm of individual observed follow-up time in person-years was included as an offset in these models to account for differences in person-time at risk. Cost outcomes were analyzed using linear regression models and reported regression coefficients as mean cost differences per person-year (PY) with 95% CI. In addition to post-COVID exposure status, all regression models adjusted for continuous age, sex, ordinal ECI, and severity of first incident COVID-19.

Individuals with no HCRU events, sick leave days, or SHI-borne costs during follow-up were retained in all analyses and contributed zero to the respective outcome. Zero values were treated as valid observed outcomes rather than as missing data.

We stratified all HCRU and cost analyses for 365-day, 730-day, and any follow-up; for the sensitivity analyses, we used a stricter censoring rule in the control cohort based on newly occurring diagnosed health outcomes associated with post-COVID.

### Generative AI and AI-Assisted Technologies

Generative AI was used in an assistive capacity to check logical consistency throughout the manuscript. It was also used to check spelling, grammar, and tone. For this purpose, ChatGPT was accessed via Shoulders Software (https://shoulde.rs) in May 2026. Any suggestions provided by AI were reviewed for accuracy before undergoing further revision by the authors.

## RESULTS

### Study Population

Of the 25 554 initially identified incident post-COVID patients, 69 (0.27%) were excluded from follow-up analyses because no suitable controls could be identified. The final matching ratio achieved was 1:1.95. As expected with exact matching, age, sex, the ECI, and acute COVID-19 severity were identically distributed between exposure and control groups (**Table 1**). Some differences remained with respect to the baseline prevalence of risk factors for severe COVID-19 (76.6% vs 72.3%). In addition, the proportion of individuals with a COVID-19 reinfection during follow-up was slightly higher in the exposure group than in the matched controls (19.5% vs 13.0%).

**Table 1.** Baseline Characteristics of Post-COVID Cases and Controls

	**Post-COVID** (**Pre-matching)**		**Post-COVID (Matched)**		**Post-COVID–Free (Matched)**	
**N**	**%**	**N**	**%**	**N**	**%**	
All	25 554	100.0	25 485	100.0	49 671	100.0
Season of first diagnosed COVID-19 episode						
2021-2022	21 064	82.4	21 004	82.4	40 932	82.4
2022-2023	3725	14.6	3718	14.6	7262	14.6
2023-2024	765	3.0	763	3.0	1477	3.0
Age group (years)						
0-5	56	0.2	56	0.2	109	0.2
6-11	222	0.9	220	0.9	434	0.9
12-17	463	1.8	463	1.8	896	1.8
18-39	6169	24.1	6154	24.1	12 019	24.2
40-59	12 294	48.1	12 262	48.1	23 943	48.2
≥60	6350	24.8	6330	24.8	12 270	24.7
Sex						
Female	15 458	60.5	15 425	60.5	30 082	60.6
Male	10 096	39.5	10 060	39.5	19 589	39.4
Severity of first acute diagnosed COVID-19 episode						
Nonhospitalized	23 850	93.3	23 844	93.6	46 629	93.9
Hospitalized	1393	5.5	1387	5.4	2625	5.3
Hospitalized + ICU	311	1.2	254	1.0	417	0.8
Elixhauser Comorbidity Index						
0	5285	20.7	5282	20.7	10 343	20.8
1	6059	23.7	6052	23.7	11 860	23.9
2	4683	18.3	4675	18.3	9136	18.4
3	3294	12.9	3282	12.9	6385	12.9
4	2210	8.6	2190	8.6	4256	8.6
≥5	4023	15.7	4004	15.7	7691	15.5
COVID-19 vaccination prior to first diagnosed COVID-19						
Yes	4826	18.9	4815	18.9	8976	18.1
No	20 728	81.1	20 670	81.1	40 695	81.9
Specialist group of first COVID-19 diagnosing physician						
General practitioner	23 267	91.1	23 233	91.2	44 705	90.0
Pediatrician	299	1.2	299	1.2	667	1.3
Cardiologist	38	0.1	38	0.1	28	0.1
Pulmonologist	136	0.5	136	0.5	77	0.2
Psychotherapist/ psychiatrist	n < 10		n < 10		n < 10	
Rehabilitation physician	n < 5		n < 5		n < 5	
Other outpatient specialist	624	2.4	621	2.4	1395	2.8
Unclear outpatient diagnosis	450	1.8	448	1.8	748	1.5
Inpatient diagnosis	729	2.9	699	2.7	2038	4.1
Specialist group of post-COVID diagnosing physician						
General practitioner	14 787	57.9	14 758	57.9	-	-
Pediatrician	259	1.0	259	1.0	-	-
Cardiologist	1978	7.7	1974	7.7	-	-
Pulmonologist	4130	16.2	4119	16.2	-	-
Psychotherapist/ psychiatrist	297	1.2	297	1.2	-	-
Rehabilitation physician	62	0.2	62	0.2	-	-
Other outpatient specialist	2948	11.5	2933	11.5	-	-
Unclear outpatient diagnosis	532	2.1	528	2.1	-	-
Inpatient diagnosis	561	2.2	555	2.2	-	-
Length of individual follow-up						
< 365 days	3123	12.2	3115	12.2	6110	12.3
365-729 days	5176	20.3	5168	20.3	10 150	20.4
≥730 days	17 255	67.5	17 202	67.5	33 411	67.3
COVID-19 reinfection during follow-up						
Yes	4978	19.5	4966	19.5	6464	13.0
No	20 576	80.5	20 519	80.5	43 207	87.0
No. of COVID-19 reinfections during follow-up						
1	4184	16.4	4173	16.4	5601	11.3
2	588	2.3	587	2.3	633	1.3
≥3	206	0.8	206	0.8	230	0.5

Abbreviation: ICU  intensive care unit.

^a^Table does not account for weighting.

### Healthcare Resource Utilization and Sick Leave

In the total matched population, individuals with incident post-COVID showed increased sick leave and HCRU across all HCRU categories compared with matched controls (**Table 2**; for detail see **Supplementary Table C**) after 365 days of follow-up, although some stratified comparisons showed lower utilization in the post-COVID group. Sick leave days exhibited the most pronounced mean differences, with an overall difference of almost +26.5 days over 1 year (post-COVID: 46.1 days [SD, 0.6]; controls: 19.6 days [SD, 0.2]). Among adults aged 40 to 59 years, the difference was particularly large, exceeding +33 days over 365 days (post-COVID: 58.0 days [SD, 0.9]; controls: 24.4 days [SD, 0.4]). Sick leave days were also notably higher among individuals with a high risk for severe COVID-19 disease (post-COVID: 51.2 days [SD, 0.7]; controls: 22.7 days [SD, 0.3]; mean difference, +28.5 days) compared with those without a prevalent risk condition (post-COVID: 29.7 days [SD, 0.9]; controls: 11.5 days [SD, 0.3]; mean difference, +18.1 days). Stratified by severity of first incident COVID-19 infection, post-COVID patients who were hospitalized during the acute phase showed the largest difference in sick leave days compared with controls (post-COVID: 47.7 days [SD, 3.0]; controls: 20.6 days [SD, 1.5]; mean difference, +27.1 days).

**Table 2.** Mean Differences in Healthcare Resource Utilization and Sick Leave Days of Incident Post-COVID Patients and Post-COVID-Free Matched Controls Over 365-Day Follow-up, Germany, 2021-2025

	**No. of Individuals**		**Mean Difference**								
**Post-COVID**	**Post-COVID– Free**	**Sick Leave Days**	**Outpatient Physician Contacts**	**Outpatient ED Visits**	**Hospitalizations**	**Hospitalizations without ICU Admission**	**Hospitalizations with ICU Admission**	**Hospital Days**	**Drug Prescriptions**	**Medical Aid Prescriptions**	
Total population	23 046	43 734	26.5	6.9	0.1	0.1	0.1	0.0	0.6	1.8	0.1
Age group											
0-5 years	56	109	0.0	2.4	0.4	-0.1	-0.1	0.0	-0.1	1.1	0.0
6-11 years	201	392	0.0	3.3	0.1	0.1	0.1	0.0	0.5	2.0	0.1
12-17 years	424	812	0.5	5.3	0.1	0.1	0.1	0.0	0.5	1.6	0.0
18-39 years	5655	10 857	23.4	7.3	0.1	0.1	0.1	0.0	0.3	1.9	0.1
40-59 years	11 054	21 029	33.6	7.2	0.1	0.1	0.1	0.0	0.8	1.9	0.2
≥60 years	5656	10 680	18.8	6.2	0.0	0.1	0.1	0.0	0.6	1.5	0.0
Prevalent risk condition											
Yes	17 610	31 498	28.5	6.5	0.1	0.1	0.1	0.0	0.5	1.1	0.0
No	5436	12 300	18.1	5.9	0.1	0.1	0.1	0.0	0.5	1.6	0.1
Severity of first acute diagnosed COVID-19 episode											
Nonhospitalized	21 520	41 086	26.4	7.1	0.1	0.1	0.1	0.0	0.8	2.0	0.1
Hospitalized	1280	2303	27.1	4.2	0.1	-0.1	0.0	-0.1	-4.3	-2.9	-0.6
Hospitalized + ICU	246	345	24.6	-10.8	-0.1	-0.1	0.0	-0.1	-1.0	-15.6	-4.0

Abbreviations: Diff, mean difference; ED, emergency department; ICU, intensive care unit.

For all details on mean and standard deviation by group, see **Supplementary Table C**.

Large and consistent mean differences were observed for outpatient physician contacts. Overall, mean outpatient physician contacts were higher over 365 days of follow-up (post-COVID: 25.8 contacts [SD, 0.1]; controls: 18.9 contacts [SD, 0.1]; mean difference, +6.9 contacts). The largest differences were among adults aged 18-39 years (post-COVID: 21.0 contacts [SD, 0.2]; controls: 13.7 contacts [SD, 0.1]; mean difference, +7.3 contacts). Moreover, there was a large negative mean difference in outpatient physician contacts for patients hospitalized in the ICU (post-COVID: 34.4 contacts [SD, 1.7]; controls: 45.2 contacts [SD, 3.4]; mean difference, −10.8 contacts).

Overall mean differences were only slightly positive for ED visits (post-COVID: 0.3 visits [SD, 0.0]; controls: 0.2 visits [SD, 0.0]; mean difference, +0.1 visits), all hospitalizations (post-COVID: 0.4 hospitalizations [SD, 0.0]; controls: 0.3 hospitalizations [SD, 0.0]; mean difference, +0.1 hospitalizations), and hospitalizations without ICU admission (post-COVID: 0.3 hospitalizations [SD, 0.0]; controls: 0.2 hospitalizations [SD, 0.0]; mean difference, +0.1 hospitalizations). There was no overall difference in hospitalizations with ICU admission (post-COVID: 0.1 hospitalizations [SD, 0.0]; controls: 0.1 hospitalizations [SD, 0.0]; mean difference, +0.0 hospitalizations).

Overall, hospital days were slightly higher in the post-COVID group (2.8 days [SD, 0.1]) compared with the post-COVID–free control group (2.2 days [SD, 0.1]; mean difference, +0.6 days). Stratified by severity of the acute COVID-19 phase, there were negative mean differences in hospital days for those hospitalized during the acute COVID-19 phase (post-COVID: 7.7 days [SD, 0.6]; controls: 12.0 days [SD, 0.8]; mean difference, -4.3 days) and for those hospitalized with an ICU admission during the acute phase (post-COVID: 14.2 days [SD, 2.5]; controls: 15.3 days [SD, 2.1]; mean difference, -1.0 days).

Mean number of prescriptions was higher overall for post-COVID (post-COVID: 13.8 prescriptions [SD, 0.1]; controls: 12.0 prescriptions [SD, 0.1]; mean difference, +1.8 prescriptions). Prescriptions were higher in the control group for patients hospitalized (post-COVID: 28.0 prescriptions [SD, 0.8]; controls: 31.0 prescriptions [SD, 0.7]; mean difference, -2.9 prescriptions) and patients who were hospitalized in the ICU (post-COVID: 35.6 prescriptions [SD, 2.5]; controls: 51.2 prescriptions [SD, 4.2]; mean difference, -15.6 prescriptions). Overall, medical aid prescriptions were only slightly higher in the post-COVID group (post-COVID: 1.4 medical aid prescriptions [SD, 0.0]; controls: 1.3 medical aid prescriptions [SD, 0.1]; mean difference, +0.1 medical aid prescriptions).

Overall, positive mean differences remained across sectors and strata in the 730-day follow-up period (**Supplementary Table D**).

Inferential analyses showed HCRU rates per person-year of post-COVID patients were significantly increased across all main domains and follow-up periods (**Figure 2**). Consistent with the descriptive analyses, the highest relative differences were observed for outpatient physician contacts and sick leave days. Over 365 days, outpatient physician contacts (incidence rate ratio [IRR], 1.39; 95% CI, 1.38-1.39), hospitalizations (IRR, 1.35; 95% CI, 1.31-1.38), drug prescriptions (IRR, 1.13; 95% CI, 1.12-1.13), and medical aid prescriptions (IRR, 1.08; 95% CI, 1.07-1.08) were all significantly increased. Similar patterns were observed over 730 days (outpatient physician contacts: IRR, 1.32 [95% CI, 1.31-1.32]; hospitalizations: IRR, 1.22 [95% CI, 1.19-1.24]; drug prescriptions: IRR, 1.10 [95% CI, 1.10-1.11]; medical aids: IRR, 1.09 [95% CI, 1.07-1.10]) and when considering any follow-up period (IRR, 1.31 [95% CI, 1.30-1.31], IRR, 1.22 [95% CI, 1.20-1.24], IRR, 1.12 [95% CI, 1.11-1.12], and IRR, 1.09 [95% CI, 1.08-1.09], respectively).

**Figure 2.**
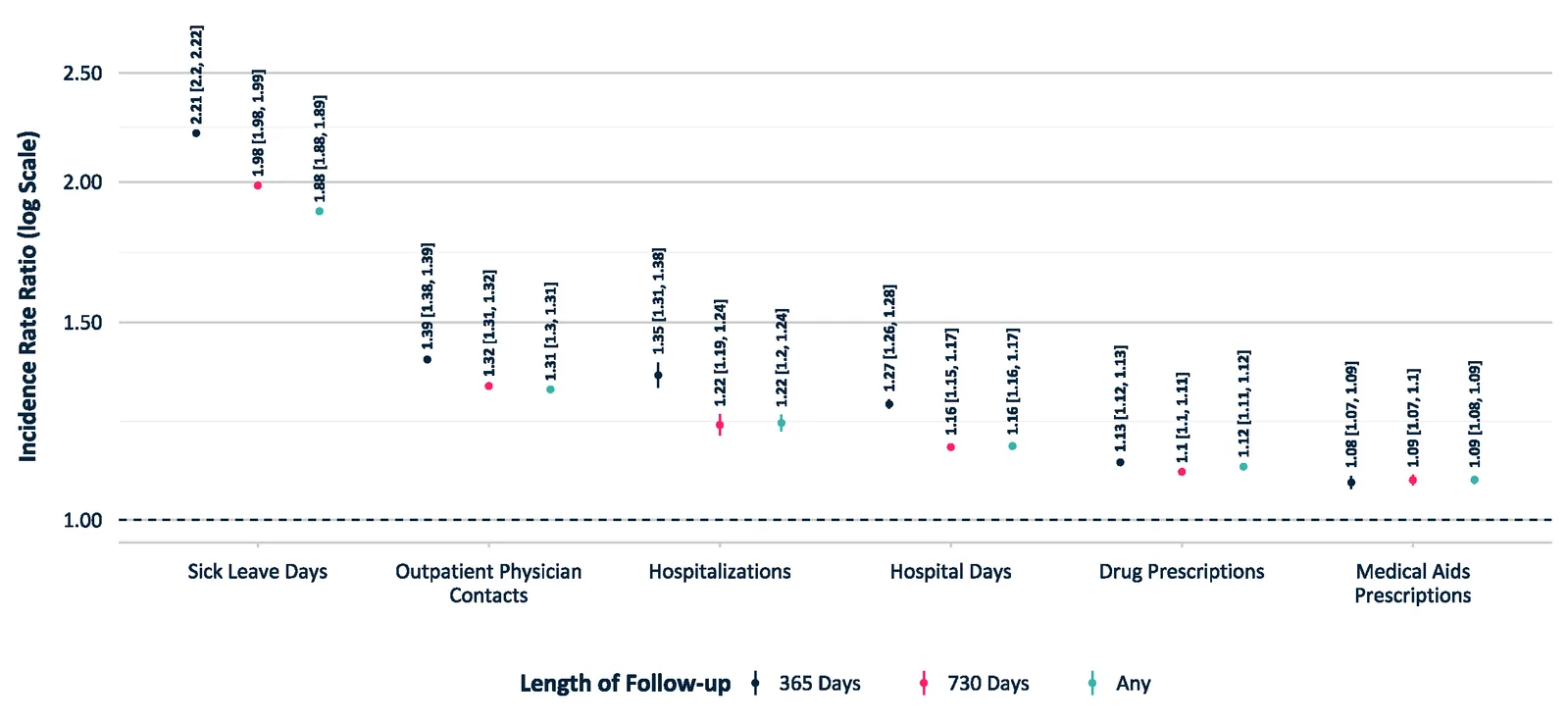
Relative Healthcare Resource Utilization and Sick Leave Rates of Post-COVID vs Post-COVID–Free Controls Over 365 Days, 730 Days, and Any Follow-up Period, Germany, 2021-2025 Note: 95% confidence intervals given in brackets.

Furthermore, compared with the main analysis, the sensitivity analysis yielded larger differences in HCRU, where the post-COVID group had higher HCRU compared with the post-COVID–free group. This finding was consistent across all HCRU parameters and follow-up periods (**Supplementary Tables G and H** and **Supplementary Figure A**).

### SHI-Borne Healthcare and Sickness Benefit Costs

In the total matched population, mean excess total SHI-borne costs of post-COVID patients amounted to €1806 per case over 365 days, although some stratified comparisons showed lower costs in the post-COVID group (**Table 3**; for detail see **Supplementary Table E**). Among age groups, the largest differences occurred in adults aged 40 to 59 years (post-COVID: €5847.2 [SD, €120.8]; post-COVID–free controls: €3775.5 [SD, €90.5]; mean difference, +€2071.7).

**Table 3.** Mean Differences in SHI-Borne Direct Healthcare and Sickness Benefit Costs of Incident Post-COVID Patients and Post-COVID–Free Matched Controls Over 365-Day Follow-up, Germany, 2021-2025

	**SHI-Borne Costs (€)**		**Mean Difference**					
**Post-COVID**	**Post-COVID–Free**	**Total Costs**	**Sickness Benefits**	**Outpatient Costs**	**Inpatient Costs**	**Prescription Drug Costs**	**Prescription Medical Aid Costs**	
Total population	23 046	43 734	1806.2	1076.9	392.5	283.3	48.7	4.8
Age group, years								
0-5	56	109	−137.4	0.0	51.9	−160.3	4.5	-33.5
6-11	201	392	506.1	0.0	197.5	270.4	67.8	−29.6
12-17	424	812	659.1	0.2	244.3	56.8	419.8	−62.0
18-39	5655	10 857	1588.9	914.0	389.9	51.8	237.2	−3.8
40-59	11 054	21 029	2071.7	1363.6	397.0	390.1	−92.4	13.3
≥60	5656	10 680	1682.3	802.1	418.3	332.6	124.7	4.5
Prevalent risk condition								
Yes	17 610	31 498	1698.8	1190.3	383.6	215.1	−74.5	−15.6
No	5436	12 300	1489.1	648.0	294.7	290.8	220.9	34.6
Severity of first acute diagnosed COVID-19 episode								
Nonhospitalized	21 520	41 086	1975.1	1068.9	401.7	352.2	140.4	11.9
Hospitalized	1280	2303	−1826.5	1063.0	300.7	−1631.2	−1469.5	−89.6
Hospitalized + ICU	246	345	−4320.7	1243.2	−779.1	−808.7	−3036.0	−940.1

Abbreviations: Diff, mean difference; SHI, statutory health insurance.

Over 365 days of follow-up, post-COVID patients with and without prevalent risk conditions for severe COVID-19 incurred higher total healthcare costs than their matched controls, but the cost differences were larger among individuals with risk conditions (post-COVID: €6758.5 [SD, €105.2]; controls: €5069.7 [SD, €83.4]; mean difference, +€1698.8).

When stratified by acute COVID-19 severity, nonhospitalized patients incurred higher excess costs in the 365-day follow-up (post-COVID: €5322.8 [SD, €81.9]; controls: €3347.7 [SD, €47.5]; mean difference, +€1975.1). By contrast, mean costs were lower among post-COVID patients than controls in the hospitalized subgroup (post-COVID: €11 450.2 [SD, €567.1]; controls: €13 276.8 [SD, €708.2]; mean difference, -€1826.5) and in the ICU subgroup (post-COVID: €20 544.2 [SD, €2468.4]; controls: €24 864.9 [SD, €2897.6]; mean difference, -€4320.7).

Overall, post-COVID patients experienced substantially higher sickness benefits during the first year after post-COVID diagnosis (post-COVID: €1459.5 [SD, €31.7]; controls: €382.6 [SD, €11.3]; mean difference, +€1076.9). Excess sickness benefit costs were most pronounced among individuals aged 40 to 59 years (post-COVID: €1870.9 [SD, €50.9]; controls: €507.3 [SD, €18.5]; mean difference, +€1,363.6) and those who were hospitalized in the ICU during the acute infection (post-COVID: €3482.4 [SD, €514.6]; controls: €2239.2 [SD, €391.0]; mean difference, +€1243.2).

Furthermore, excess outpatient costs amounted to €393.5 per case (post-COVID: €1358.2 [SD, €12.0]; controls: €965.7 [SD, €8.9]]; excess inpatient costs to €283.3 per case (post-COVID: €1596.2 [SD, €49.7]; controls: €1312.9 [SD, €34.5]]; excess prescription costs to +€48.7 per case (post-COVID: €1192.7 [SD, €46.5]; controls: €1144.0 [SD, €39.6]); and medical aid costs to €4.8 per case (post-COVID: €188.0 [SD, €7.9]; controls: €183.2 [SD, €7.8]).

Over the 730-day follow-up period, SHI-borne costs remained higher overall among post-COVID patients, although stratum-specific differences varied in magnitude and direction. Mean excess SHI-borne costs of post-COVID patients totaled €2434.3 (post-COVID: €10,362.9 [SD, €161.2]; controls: €7928.6 [SD, €123.6]) and excess sickness benefits amounted to €1410.4 (post-COVID: €2173.9 [SD, €49.6]; controls: €763.6 [SD, €20.5]) in the 730-day follow-up period (**Supplementary Table F**).

In inferential analyses, cost differences (per PY) of post-COVID patients were significantly higher across all follow-up periods for all cost domains except for prescription drugs and medical aids (**Figure 3**). On average, total SHI-borne costs were higher by €1663.41 per PY (95% CI, €1457.08-€1869.75) over 365-day, €1224.34 per PY (95% CI, €1016.63-€1432.05) over 730-day, and €1224.87 (95% CI, €1052.01-€1397.73) over any follow-up.

**Figure 3.**
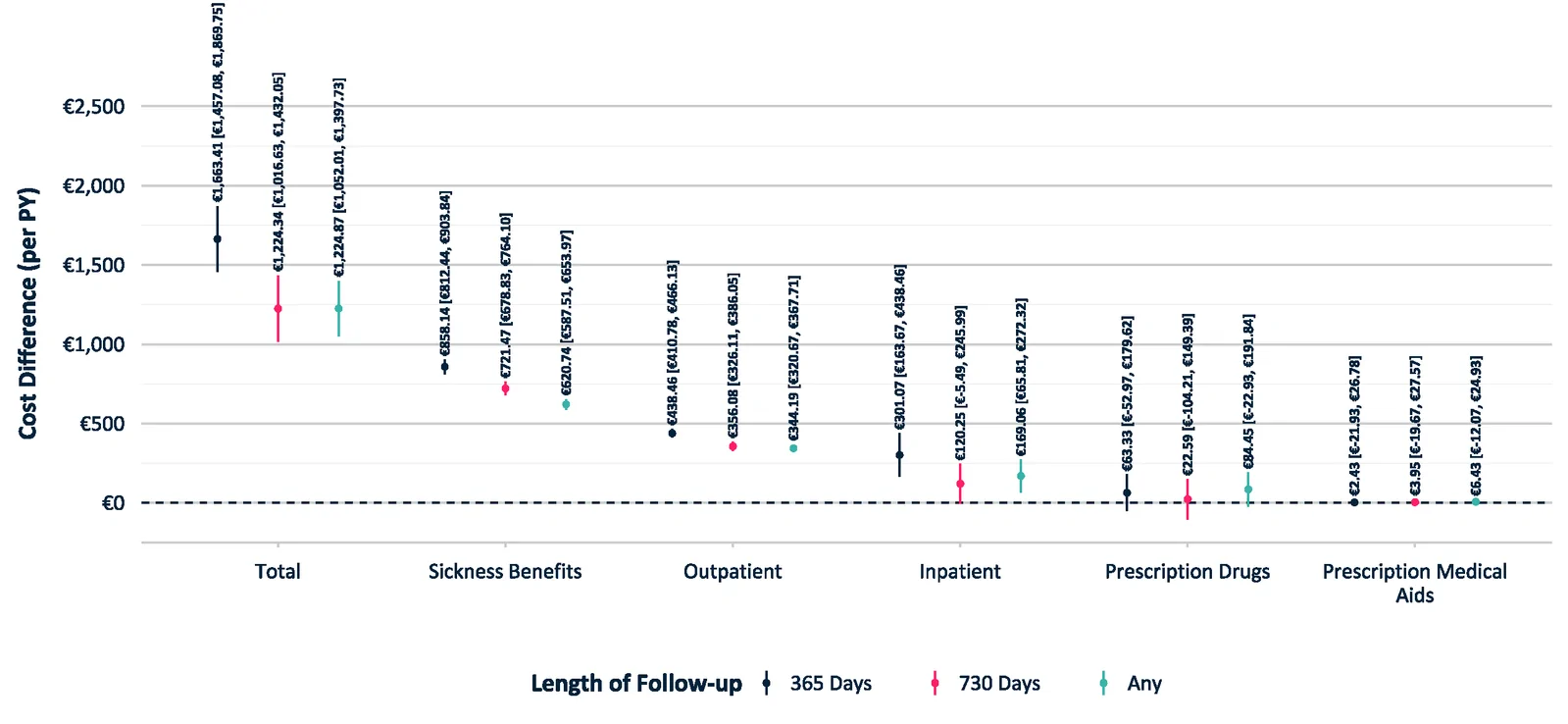
Mean Differences in SHI-Borne Direct Healthcare and Sickness Benefit Costs (€) of Incident Post-COVID Patients and Post-COVID–Free Matched Controls Over 365-Day Follow-up, Germany, 2021-2025 Abbreviation: PY, person-year.

Outpatient costs were significantly higher by €438.46 per PY (95% CI, €410.78-€466.13) over 365 days (730 days: €356.08 per PY; 95% CI, €326.11-€386.05; any: €344.19 per PY; 95% CI, €320.67-€367.71), inpatient costs were significantly higher by €301.07 per PY (95% CI, €163.67-€438.46) over 365 days (730 days: €120.25 per PY; 95% CI, -€5.49-€245.99; any: €169.06 per PY; 95% CI, €65.81-€272.32) and sickness benefit costs were significantly higher by €858.14 per PY (95% CI, €812.44-€903.84) over 365 days (730 days: €721.47; 95% CI, €678.83-€764.10; any: €620.74 per PY; 95% CI, €587.51-€653.97).

Prescription drug costs (365 days: €63.33 per PY; 95% CI, -€52.97-€179.62; 730 days: €22.59; 95% CI, -€104.21-€149.39; any €84.45; 95% CI, -€22.93-€191.84) and medical aid costs (365 days: €2.43 per PY; 95% CI, -€21.93-26.78; 730 days: €3.95; 95% CI, -€19.67-€27.57; any €6.43; 95% CI, -€12.07-€24.93) were higher in the post-COVID group, but the differences were not statistically significant.

The sensitivity analyses yielded larger cost differences than the main analyses. This finding was consistent across all cost parameters and follow-up periods (**Supplementary Tables I and J** and **Supplementary Figure B**).

## DISCUSSION

In this analysis, incident documented post-COVID was associated with consistently higher resource utilization, with the largest differences observed for sick leave and outpatient physician contacts. Over 365 days, patients with post-COVID had approximately 27 additional sick leave days and 7 more outpatient physician contacts compared with controls. Post-COVID was also associated with higher total SHI-borne costs of €1806 per case compared with controls, including €1077 in mean excess sickness benefit costs over 365 days. Overall, the economic burden was greatest among working-age adults aged 18 to 59 years.

We found a strong association between post-COVID and increased sick leave days. The largest excess burden was apparent for individuals aged 40 to 59 years, with +34 additional sick days compared with controls and an additional +€1364 in sickness benefits compared with controls during the 365-day follow-up period. Furthermore, excess sick leave days for the age group 40 to 59 years were +47 in the 730-day follow-up period, indicating that the excess burden of sick leave associated with post-COVID persisted over longer follow-up. These results are consistent with prior evidence that the economic burden of post-COVID is high in the working-age population both in terms of costs^20^ and impaired work ability,^24^ with effects generally persisting over time.^21^

Furthermore, sick leave days and related costs were higher among patients with underlying medical conditions. This is broadly consistent with previous studies showing greater risk of prolonged work absence among patients with more severe disease^23^ or greater baseline vulnerability in terms of chronic medical conditions.^25^ However, these studies also often find a relationship between advanced age and longer sick leave. This holds only to a certain extent in our findings, as excess sick leave was longer for those aged 40 to 59 years (+34 days) compared with those aged 18 to 39 years (+23 days), but excess sick leave was shorter on average for individuals 60 years or older (+19). However, this average likely reflects the inclusion of retirees, making sick leave less interpretable for this age group.

We also found that post-COVID was associated with increased outpatient physician contacts, whereas differences in hospitalizations and other sectors were smaller and, in some severity-stratified comparisons, were lower in the post-COVID group. The increased outpatient use is consistent with prior studies reporting increased outpatient utilization among patients with post-COVID or long COVID.^17,18^ In particular, lower outpatient physician contacts, hospital days, and prescription use were observed among some patients who had been hospitalized or admitted to the ICU during acute COVID-19. The more modest overall differences observed in inpatient care and prescription use, including lower utilization in some severity strata, suggest considerable heterogeneity in these outcomes. These patterns may reflect residual differences between groups and should be interpreted cautiously, particularly given the limited published evidence from Germany for comparison.

In terms of total costs, we found average total SHI-borne costs of approximately €5795 per post-COVID case over a 365-day follow-up period. Given that our estimate of total costs included mean sickness benefits of €1460, the resulting difference of €4335 is consistent with the limited cost evidence from Germany, which estimated a mean of approximately €4600 per post-COVID case without sickness benefits, with varying costs by severity and length of inpatient stay.^14^

Mean excess total SHI-borne costs were €1806 over 365 days, which corresponds to a modest relative increase compared with controls. Although this estimate is lower than some published estimates from other countries, the direction of effect is broadly consistent with prior evidence that post-COVID is associated with higher healthcare spending.^18–20^ Direct comparison across studies remains difficult because case definitions, follow-up periods, comparator groups, and costing frameworks differ substantially. In our study, post-COVID was identified based on ICD-10 codes and only diagnoses documented during the post-acute phase, starting 90 days after the first documented COVID-19 diagnosis, were considered. This approach applied a more restrictive temporal definition of post-COVID. In contrast, other studies rely on definitions that conflate long COVID and post-COVID,^18^ which may lead to higher overall utilization estimates than our more restrictive definition. Ultimately, within our study, excess costs were highest among adults aged 40 to 59 years, consistent with the age pattern observed for sick leave and sickness benefit costs and suggesting that the measured burden was concentrated in working-age individuals.

However, stratification by acute SARS-CoV-2 infection severity yielded results that were not in line with prior expectations. Over 365 days of follow-up, mean cost differences relative to controls were +€1975 among patients with post-COVID who were not hospitalized during the acute phase, but -€1827 among those hospitalized and -€4321 among those admitted to the ICU. This pattern contrasts with prior studies, which generally report higher HCRU and costs with greater acute COVID-19 severity or hospitalization.^14,19,33^ The unexpected findings appear to be driven largely by inpatient utilization: although the proportion of hospitalizations was similar between post-COVID and control groups due to our matched design, mean hospital days were higher in the post-COVID group only among patients not hospitalized during the acute phase (+0.8 days), whereas mean hospital days were higher in controls among those hospitalized during the acute phase (+4.3 days) and among those admitted to the ICU (+1.0 days). These findings should be interpreted with caution as they may reflect confounding by unknown factors that might influence the likelihood of receiving a post-COVID diagnosis. For example, we did not distinguish between hospitalizations primarily for COVID-19 and hospitalizations with secondary COVID-19,^31^ which may be an unmeasured confounder of apparent associations between acute infection severity, diagnosis (or nondiagnosis) of post-COVID, and HCRU. Furthermore, because acute COVID-19 severity was included in the matching criteria, our estimates reflect the additional HCRU, sick leave, and SHI-borne costs associated with documented post-COVID among individuals with comparable acute disease severity. They should therefore not be interpreted as the full downstream burden attributable to COVID-19 and all its sequelae.

Finally, in the overall population, we found that HCRU, sick leave, and costs of post-COVID patients remained higher over the longer 730-day period, indicating a sustained burden beyond the first year. Mean cumulative excess total costs increased from €1806 per case over 365 days to €2434 per case over 730 days. Thus, the cumulative excess burden continued to increase over time, although the additional excess costs accrued during the second year were substantially lower than those accrued during the first year (€628 vs €1806). This pattern is echoed in the literature, where patients with post-COVID had higher HCRU for up to 2 years following diagnosis of COVID-19,^19^ with the highest utilization rates often in the first few months following diagnosis of post-COVID or related syndromes.^18^

This study has several limitations. First, SHI claims data are designed for billing, not research, and lack clinical detail such as symptom severity, laboratory results, imaging, functional status, and patient-reported outcomes. Our analyses therefore relied on diagnosis codes and healthcare contacts, which may be affected by coding errors and differences in documentation across providers and over time. Second, both COVID-19 and post-COVID may have been incompletely captured. Some individuals may have had undocumented acute infection but later received a post-COVID diagnosis, whereas others with persistent symptoms may never have been coded as post-COVID and may therefore have been classified as post-COVID-free controls. To the extent that such exposure misclassification occurred nondifferentially, it would be expected to attenuate observed between-group differences in HCRU, sick leave, and SHI-borne costs. The larger differences observed in the sensitivity analysis were consistent with possible attenuation of the primary estimates due to such misclassification. Third, documented COVID-19 reinfections during follow-up were more frequent among post-COVID cases than among matched controls (19.5% vs 13.0%). Because reinfections occurred after cohort entry, they were not included as baseline covariates in the primary regression models. The observed difference may reflect differences in reinfection risk, residual differences in factors not captured by the matching procedure, or differential ascertainment related to healthcare contacts. Regardless of the underlying mechanism, subsequent reinfections may have contributed to some of the observed between-group differences in HCRU, sick leave, and SHI-borne costs. Fourth, recent analyses by the Zentralinstitut indicate that, in Q2 2024, approximately 33% of individuals with a post-COVID diagnosis had no documented COVID-19 diagnosis in the preceding 4 years.^34^ These cases may represent individuals who self-identified as having had COVID-19 without a formal test or diagnosis. This indicates that our cohort likely excluded some true prior infections, which may lead us to underestimate post-COVID incidence and introduce selection bias if documented cases differ systematically from undocumented infections. Finally, our anonymized dataset comes from 20 nationwide SHI funds. Therefore, our data include neither privately insured individuals, such as civil servants, nor individuals insured at other SHI funds. Because individuals self-select into SHI funds and, to some extent, private insurance, our results may be less generalizable to these populations.

## CONCLUSIONS

In this real-world study based on anonymized German claims data, incident post-COVID was associated with persistent increases in HCRU, sick leave, and SHI-borne direct costs, with the greatest excess burden seen in sick leave and outpatient physician contacts, particularly among working-age adults. These findings suggest that much of the long-term economic burden of post-COVID may fall on outpatient care and work productivity rather than inpatient care. Targeted prevention strategies are needed to mitigate long-term impacts on HCRU and especially labor productivity.

### Disclosures

D.G., K.B., and J.D. are employees of Vandage GmbH (Bielefeld, Germany). D.G. is a shareholder of Vandage. Vandage received funding from Pfizer Pharma GmbH (Berlin, Germany) to conduct the present study. Vandage received further payments from GlaxoSmithKline GmbH & Co. KG (Munich, Germany), Janssen-Cilag GmbH (Neuss, Germany), MSD Sharp & Dohme GmbH (Munich, Germany), Moderna Germany GmbH (Munich, Germany), Sanofi-Aventis Deutschland GmbH (Berlin, Germany), and consulting fees and grants from AOK Rheinland/Hamburg (Düsseldorf, Germany), BARMER (Berlin, Germany), DAK-Gesundheit (Hamburg, Germany), Federal Joint Committee (Berlin, Germany), and Techniker Krankenkasse (Hamburg, Germany). E.F., A.K., J.N., and J.B. are employees of Pfizer and may own stock or stock options. P.R. is an employee of Jena University Hospital (Jena, Germany) and received lecture and/or consulting fees from Pfizer Pharma GmbH (Berlin, Germany), BMS (Munich, Germany), Gilead (Munich, Germany), CSL Seqirus (Munich, Germany), AstraZeneca (Hamburg, Germany), and AbbVie (Wiesbaden, Germany). E.OiK. was an employee of Pfizer at the time of the study but not at publication.

### Ethics Statement

The study was conducted according to the Declaration of Helsinki of 1964. Following German data protection legislation, ethical approval and informed consent were not required for this study, as patient data were completely anonymized by the data provider before being made available for research. Ethical approval and informed consent were therefore waived for this study.

### Declaration of Generative AI and AI-assisted Technologies in the Writing Process

Generative AI was used in an assistive capacity to check logical consistency throughout the manuscript. It was also used to check spelling, grammar, and tone. For this purpose, ChatGPT was accessed via Shoulders Software in May 2026. Any suggestions provided by AI were reviewed for accuracy before undergoing further revision by D.G. and K.B.

## Supporting information

Online Supplementary Material
