# Supplementary material for: Economic Burden of Post-COVID Syndrome in Germany Between 2021 and 2025: A Statutory Health Insurance Claims Data Analysis
Source: J Health Econ Outcomes Res. 2026 Oct 21;13(2):178–90. doi: 10.36469/001c.170196 (PMC13693698; doi:10.36469/001c.170196)
Supplement: Supplementary file 1 [file jheor_2026_13_2_170196_368095.pdf]

Economic Burden of COVID-19 and Post-COVID Syndrome in Germany Between 2021 and 2025: A Statutory Health Insurance Claims Data Analysis

Supplementary Material

Tables

Table A: Operational definitions of covariates and other descriptive variables 3

Table B: Age distribution of matched post-COVID cases by risk condition for severe COVID-19 and severity of the first acute diagnosed COVID-19 episode 7

Table C: Mean (SD) healthcare resource utilization and sick leave days of incident post-COVID patients and post-COVID-free matched controls over 365-day follow-up, Germany, 2021–2025......... 8

Table D: Mean (SD) healthcare resource utilization and sick leave days of incident post-COVID patients and post-COVID-free matched controls over 730-day follow-up, Germany, 2021–2025....... 11

Table E: Mean (SD) SHI-borne direct healthcare and sickness benefit costs in € of incident post-COVID patients and post-COVID-free matched controls over 365-day follow-up, Germany, 2021–2025....... 14

Table F: Mean (SD) SHI-borne direct healthcare and sickness benefit costs in € of incident post-COVID patients and post-COVID-free matched controls over 730-day follow-up, Germany, 2021–2025....... 17

Table G: Mean (SD) healthcare resource utilization and sick leave days of incident post-COVID patients and post-COVID-free matched controls over 365-day follow-up, Germany, 2021–2025, sensitivity analyses with newly-occurring diagnosed post-COVID-related health outcomes as censoring events 20

Table H: Mean (SD) healthcare resource utilization and sick leave days of incident post-COVID patients and post-COVID-free matched controls over 730-day follow-up, Germany, 2021–2025, sensitivity analyses with newly-occurring diagnosed post-COVID-related health outcomes as censoring events 23

Table I: Mean (SD) SHI-borne direct healthcare and sickness benefit costs in € of incident post-COVID patients and post-COVID-free matched controls over 365-day follow-up, Germany, 2021–2025, sensitivity analyses with newly-occurring diagnosed post-COVID-related health outcomes as censoring event 27

Table J: Mean (SD) SHI-borne direct healthcare and sickness benefit costs in € of incident post-COVID patients and post-COVID-free matched controls over 730-day follow-up, Germany, 2021–2025, sensitivity analyses with newly-occurring diagnosed post-COVID-related health outcomes as censoring event 30

Figures

Figure A: Relative healthcare resource utilization and sick leave rates of post-COVID compared to post-COVID-free controls over 365 days, 730 days, and any follow-up period, Germany, 2021–2025, sensitivity analyses with newly-occurring diagnosed post-COVID-related health outcomes as censoring event. 95% confidence intervals given in brackets. 26

Figure B: Differences in SHI-borne direct healthcare and sickness benefit costs per person-year between post-COVID and post-COVID-free controls over 365 days, 730 days, and any follow-up period, Germany, 2021–2025, sensitivity analyses with newly-occurring diagnosed post-COVID-related health outcomes as censoring events. 95% confidence intervals given in brackets. 33

Table A: Operational definitions of covariates and other descriptive variables

**Variable**	**Description**	**Variable type**	**Assessment window**	**Setting**	**Code type**	**Used as**	**Source**
Birth year interval	Year of birth, categorized in standardized 5-year intervals.<br>*Note: Aggregation is pre-defined by the data provider due to data privacy reasons*	Categorical	n/a	n/a	n/a	M	n/a
Age	Age (in years) at cohort entry based on 5-year birth intervals. Age is determined as follows:<br>Year of Time 0 or PED<br>MINUS first year of the 5-year birth interval<br>PLUS a random number 0 to 4<br>*Note: The same age is assigned to a Post-COVID case and its matched control(s)*	Continuous	[0, 0]	n/a	n/a	C	n/a
Age group	Age, categorized as follows:<br>• 0-5 years<br>• 6-11 years<br>• 12-17 years<br>• 18-59 years<br>• ≥60 years	Categorical	[0, 0]	n/a	n/a	C, P	n/a
Sex	Biological sex, categorized as follows:<br>• Male<br>• Female	Categorical	[0, 0]	n/a	n/a	C, M, P	n/a
Elixhauser Comorbidity Index	Elixhauser Comorbidity Index, categorized as follows:<br>• 0<br>• 1<br>• 2<br>• 3<br>• 4	Categorical	[-365, -1]	IP (P/S)<br>OP (G)	ICD	P, C	[23, 24]

Variable	Description	Variable type	Assessment window	Setting	Code type	Used as	Source
	• ≥5						
Prevalent underlying risk condition for severe COVID-19	Documented diagnosis one of the following underlying risk conditions in two different inpatient or outpatient cases:<br>• Lung diseases<br>• Cardiovascular diseases<br>• Liver diseases<br>• Kidney diseases<br>• Diabetes mellitus<br>• Obesity<br>• Neurological diseases<br>• Psychiatric diseases<br>• Down syndrome<br>• Immunodeficiency<br>• Cancer	Binary	[-365, -1]	IP (P/S)<br>OP (G)	ATC<br>ICD<br>OPS	C, P	[27, 28]
Prior COVID-19 vaccination	Documented COVID-19 vaccination.	Binary	[-365, -1]	OP	EBM	P	n/a
Severity of first incident COVID-19 disease	Severity of first incident COVID-19 disease, categorized as follows:<br>• Not hospitalized due to COVID-19 during acute phase<br>• Hospitalized due to COVID-19 w/o ICU admission during acute phase<br>• Hospitalized due to COVID-19 w/ ICU admission during acute phase	Categorical	[PED, PED+89]	OP<br>IP	ICD	C, M, P	n/a
Specialist group of first COVID-19-diagnosing physician	Specialist group of the physician who documented the first incident COVID-19 diagnosis qualifying for population entry, categorized as follows:<br>• General practitioner<br>• Pediatrician	Categorical	[PED]	OP<br>IP	n/a	P	n/a

Variable	Description	Variable type	Assessment window	Setting	Code type	Used as	Source
	• Cardiologist<br>• Pulmonologist<br>• Psychotherapist/psychiatrist<br>• Rehabilitation physician<br>• Other outpatient specialist<br>• Inpatient diagnosis						
Specialist group of Post-COVID-diagnosing physician	Specialist group of the physician who documented the Post-COVID diagnosis qualifying for exposure cohort entry, categorized as follows:<br>• General practitioner<br>• Pediatrician<br>• Cardiologist<br>• Pulmonologist<br>• Psychotherapist/psychiatrist<br>• Rehabilitation physician<br>• Other outpatient specialist<br>• Inpatient diagnosis	Categorical	[0, 0]	OP<br>IP	n/a	P	n/a
Calendar quarter of first incident COVID-19 diagnosis	Calendar quarter during which the first incident COVID-19 diagnosis qualifying for population entry was documented (i.e., PED). One category for each calendar quarter of the population entry period from Q4/2021 to Q3/2024.	Categorical	[PED]	n/a	n/a	M	n/a
Season of first incident COVID-19 diagnosis	COVID-19 season during which the first incident COVID-19 diagnosis qualifying for population entry was documented (i.e., PED), categorized as follows:<br>• Season 2021/22<br>• Season 2022/23<br>• Season 2023/24	Categorical	[PED]	n/a	n/a	C, P	n/a
Calendar quarter of cohort entry	Calendar quarter during which the individual entered the exposure/comparator group (i.e., Time	Categorical	[PED]	n/a	n/a	M	n/a

Variable	Description	Variable type	Assessment window	Setting	Code type	Used as	Source
	0). One category for each calendar quarter of the population entry period from OCT 2021 to SEP 2024.						
Season of cohort entry	COVID-19 season during which the individual entered the exposure/comparator group (i.e., Time 0), categorized as follows:<br>• Season 2021/22<br>• Season 2022/23<br>• Season 2023/24	Categorical	[PED]	n/a	n/a	P	n/a
Length of individual follow-up	Length of individual follow-up, categorized as:<br>• <365 days<br>• 365-729 days<br>• ≥730 days	Categorical	[0, censor]	n/a	n/a	P	n/a
COVID-19 reinfection (during follow-up)	Documented COVID-19 diagnosis considered as reinfection (i.e., with 90-day diagnosis-free washout before the diagnosis) during follow-up.	Binary	[0, censor]	IP (P/S)<br>OP (G)	ICD	P	n/a
No. of COVID-19 reinfections (during follow-up)	No. of documented COVID-19 diagnoses considered as reinfection (i.e., with 90-day diagnosis-free washout before the diagnosis) during follow-up, categorized as:<br>• 1 reinfection<br>• 2 reinfections<br>• ≥3 reinfections	Categorical	[0, censor]	IP (P/S)<br>OP (G)	ICD	P	n/a

General abbreviations: PED: Population Entry Date, PO: Primary Objective, SO: Secondary Objective

Care settings: D: prescription drugs, IP: inpatient, MA: medical aids, OP: outpatient; SL: sick leave

Diagnosis positions: G: confirmed (diagnosis code "G – Gesichert"), P: primary, S: secondary, V: suspected (diagnosis code "V – Verdacht")

Code types: ATC: Anatomical Therapeutic Chemical Classification System, EBM: outpatient fee, ICD: International Statistical Classification of Diseases and Related Health Problems (10th Revision, German Modification), OPS: Operations and Procedure Code

Used as: C: covariates for stratification or regression analyses, M: matching variables, P: population characterization

Table B: Age distribution of matched post-COVID cases by risk condition for severe COVID-19 and severity of the first acute diagnosed COVID-19 episode

	**Prevalent risk condition for severe COVID-19**				**Severity of first incident COVID-19 disease**					
	**Yes**		**No**		**Non-hospitalized**		**Hospitalized**		**Hospitalized + ICU**	
**N**	19,514		5,971		23,844		1,387		254	
**Age group**										
0-5 years	24	0.1%	32	0.5%	55	0.2%	n<5	n<5	0	0.0%
6-11 years	89	0.5%	131	2.2%	216	0.9%	n<5	n<5	0	0.0%
12-17 years	179	0.9%	284	4.8%	451	1.9%	12	1%	0	0.0%
18-39 years	3,568	18.3%	2,586	43.3%	5,982	25.1%	162	11.7%	10	3.9%
40-59 years	9,748	50.0%	2,514	42.1%	11,754	49.3%	420	30.3%	88	34.6%
≥60 years	5,906	30.3%	424	7.1%	5,386	22.6%	788	56.8%	156	61.4%

N: number of matched post-COVID cases; ICU: intensive care unit

Table C: Mean (SD) healthcare resource utilization and sick leave days of incident post-COVID patients and post-COVID-free matched controls over 365-day follow-up, Germany, 2021–2025

	**No. of individuals**		**Sick leave days**			**Outpatient physician contacts**			**Outpatient ED visits**		
	**Post-COVID**	**Post-COVID-free**	**Post-COVID**	**Post-COVID-free**	**Diff.**	**Post-COVID**	**Post-COVID-free**	**Diff.**	**Post-COVID**	**Post-COVID-free**	**Diff.**
**Total population**	23,046	43,734	46.1 (0.6)	19.6 (0.2)	26.5	25.8 (0.1)	18.9 (0.1)	6.9	0.3 (0.0)	0.2 (0.0)	0.1
**Age group**											
0-5 years	56	109	0.0 (0.0)	0.0 (0.0)	0.0	12.4 (1.1)	10.0 (0.8)	2.4	0.8 (0.2)	0.4 (0.1)	0.4
6-11 years	201	392	0.0 (0.0)	0.0 (0.0)	0.0	12.3 (0.7)	9.0 (0.5)	3.3	0.5 (0.1)	0.3 (0.0)	0.1
12-17 years	424	812	0.9 (0.2)	0.5 (0.1)	0.5	14.8 (0.6)	9.6 (0.4)	5.3	0.4 (0.0)	0.3 (0.0)	0.1
18-39 years	5,655	10,857	40.5 (1.0)	17.0 (0.4)	23.4	21.0 (0.2)	13.7 (0.1)	7.3	0.4 (0.0)	0.3 (0.0)	0.1
40-59 years	11,054	21,029	58.0 (0.9)	24.4 (0.4)	33.6	25.7 (0.2)	18.4 (0.1)	7.2	0.2 (0.0)	0.2 (0.0)	0.1
≥60 years	5,656	10,680	33.4 (1.1)	14.6 (0.5)	18.8	32.2 (0.3)	26.0 (0.2)	6.2	0.3 (0.0)	0.2 (0.0)	0.0
**Prevalent risk condition**											
Yes	17,610	31,498	51.2 (0.7)	22.7 (0.3)	28.5	29.1 (0.2)	22.6 (0.1)	6.5	0.3 (0.0)	0.2 (0.0)	0.1
No	5,436	12,300	29.7 (0.9)	11.5 (0.3)	18.1	15.1 (0.1)	9.2 (0.1)	5.9	0.2 (0.0)	0.2 (0.0)	0.1
**Severity of first acute diagnosed COVID-19 episode**											
Non-hospitalized	21,520	41,086	45.8 (0.6)	19.4 (0.2)	26.4	25.2 (0.1)	18.1 (0.1)	7.1	0.3 (0.0)	0.2 (0.0)	0.1
Hospitalized	1,280	2,303	47.7 (3.0)	20.6 (1.5)	27.1	34.5 (0.8)	30.3 (0.7)	4.2	0.5 (0.0)	0.4 (0.0)	0.1
Hospitalized + ICU	246	345	66.4 (8.3)	41.7 (5.9)	24.6	34.4 (1.7)	45.2 (3.4)	-10.8	0.3 (0.1)	0.4 (0.1)	-0.1
Abbreviations: Abbreviations: Abbreviations: Diff: mean difference, ED emergency department, ICU: intensive care unit, SD: standard deviation., SD: standard deviation.											

Table C: Mean (SD) healthcare resource utilization and sick leave days of incident post-COVID patients and post-COVID-free matched controls over 365-day follow-up, Germany, 2021–2025 (continued)

	**No. of individuals**		**Hospitalizations**			**Hospitalizations w/o ICU admission**			**Hospitalizations w/ ICU admission**		
	**Post-COVID**	**Post-COVID-free**	**Post-COVID**	**Post-COVID-free**	**Diff.**	**Post-COVID**	**Post-COVID-free**	**Diff.**	**Post-COVID**	**Post-COVID-free**	**Diff.**
**Total population**	23,046	43,734	0.4 (0.0)	0.3 (0.0)	0.1	0.3 (0.0)	0.2 (0.0)	0.1	0.1 (0.0)	0.1 (0.0)	0.0
**Age group**											
0-5 years	56	109	0.1 (0.1)	0.2 (0.1)	-0.1	0.1 (0.1)	0.2 (0.1)	-0.1	0.0 (0.0)	0.0 (0.0)	0.0
6-11 years	201	392	0.2 (0.0)	0.1 (0.0)	0.1	0.2 (0.0)	0.1 (0.0)	0.1	0.0 (0.0)	0.0 (0.0)	0.0
12-17 years	424	812	0.2 (0.0)	0.1 (0.0)	0.1	0.2 (0.0)	0.1 (0.0)	0.1	0.0 (0.0)	0.0 (0.0)	0.0
18-39 years	5,655	10,857	0.3 (0.0)	0.2 (0.0)	0.1	0.3 (0.0)	0.2 (0.0)	0.1	0.0 (0.0)	0.0 (0.0)	0.0
40-59 years	11,054	21,029	0.3 (0.0)	0.2 (0.0)	0.1	0.3 (0.0)	0.2 (0.0)	0.1	0.0 (0.0)	0.0 (0.0)	0.0
≥60 years	5,656	10,680	0.6 (0.0)	0.5 (0.0)	0.1	0.4 (0.0)	0.3 (0.0)	0.1	0.1 (0.0)	0.1 (0.0)	0.0
**Prevalent risk condition**											
Yes	17,610	31,498	0.4 (0.0)	0.3 (0.0)	0.1	0.4 (0.0)	0.3 (0.0)	0.1	0.1 (0.0)	0.1 (0.0)	0.0
No	5,436	12,300	0.2 (0.0)	0.1 (0.0)	0.1	0.2 (0.0)	0.1 (0.0)	0.1	0.0 (0.0)	0.0 (0.0)	0.0
**Severity of first acute diagnosed COVID-19 episode**											
Non-hospitalized	21,520	41,086	0.3 (0.0)	0.2 (0.0)	0.1	0.3 (0.0)	0.2 (0.0)	0.1	0.0 (0.0)	0.0 (0.0)	0.0
Hospitalized	1,280	2,303	0.9 (0.0)	1.0 (0.0)	-0.1	0.7 (0.0)	0.6 (0.0)	0.0	0.3 (0.0)	0.4 (0.0)	-0.1
Hospitalized + ICU	246	345	1.1 (0.1)	1.3 (0.1)	-0.1	0.0 (0.0)	0.0 (0.0)	0.0	1.1 (0.1)	1.3 (0.1)	-0.1
Abbreviations: Abbreviations: Abbreviations: Diff: mean difference, ED emergency department, ICU: intensive care unit, SD: standard deviation., SD: standard deviation.											

Table C: Mean (SD) healthcare resource utilization and sick leave days of incident post-COVID patients and post-COVID-free matched controls over 365-day follow-up, Germany, 2021–2025 (continued)

	No. of individuals		Hospital days			Drug prescriptions			Medical aid prescriptions		
	Post-COVID	Post-COVID-free	Post-COVID	Post-COVID-free	Diff.	Post-COVID	Post-COVID-free	Diff.	Post-COVID	Post-COVID-free	Diff.
**Total population**	23,046	43,734	2.8 (0.1)	2.2 (0.1)	0.6	13.8 (0.1)	12.0 (0.1)	1.8	1.4 (0.0)	1.3 (0.0)	0.1
**Age group**											
0-5 years	56	109	0.2 (0.1)	0.3 (0.1)	-0.1	8.7 (1.0)	7.6 (0.9)	1.1	0.4 (0.1)	0.4 (0.1)	0.0
6-11 years	201	392	1.3 (0.5)	0.7 (0.5)	0.5	7.7 (0.6)	5.7 (0.6)	2.0	0.7 (0.2)	0.6 (0.2)	0.1
12-17 years	424	812	1.4 (0.4)	0.9 (0.2)	0.5	5.4 (0.3)	3.8 (0.2)	1.6	0.8 (0.2)	0.8 (0.2)	0.0
18-39 years	5,655	10,857	2.0 (0.1)	1.7 (0.1)	0.3	6.9 (0.1)	5.1 (0.1)	1.9	0.6 (0.0)	0.6 (0.0)	0.1
40-59 years	11,054	21,029	2.6 (0.1)	1.8 (0.1)	0.8	12.7 (0.1)	10.9 (0.1)	1.9	1.2 (0.0)	1.0 (0.0)	0.2
≥60 years	5,656	10,680	4.3 (0.2)	3.7 (0.2)	0.6	23.7 (0.3)	22.2 (0.3)	1.5	2.6 (0.1)	2.6 (0.1)	0.0
**Prevalent risk condition**											
Yes	17,610	31,498	3.3 (0.1)	2.8 (0.1)	0.5	16.6 (0.1)	15.5 (0.1)	1.1	1.7 (0.0)	1.6 (0.0)	0.0
No	5,436	12,300	1.2 (0.1)	0.7 (0.1)	0.5	4.6 (0.1)	3.0 (0.0)	1.6	0.5 (0.0)	0.3 (0.0)	0.1
**Severity of first acute diagnosed COVID-19 episode**											
Non-hospitalized	21,520	41,086	2.4 (0.1)	1.6 (0.0)	0.8	12.8 (0.1)	10.8 (0.1)	2.0	1.2 (0.0)	1.1 (0.0)	0.1
Hospitalized	1,280	2,303	7.7 (0.6)	12.0 (0.8)	-4.3	28.0 (0.8)	31.0 (0.7)	-2.9	3.7 (0.3)	4.3 (0.3)	-0.6
Hospitalized + ICU	246	345	14.2 (2.5)	15.3 (2.1)	-1.0	35.6 (2.5)	51.2 (4.2)	-15.6	7.4 (1.2)	11.4 (1.8)	-4.0
Abbreviations: Abbreviations: Diff: mean difference, ED emergency department, ICU: intensive care unit, SD: standard deviation., SD: standard deviation.											

Table D: Mean (SD) healthcare resource utilization and sick leave days of incident post-COVID patients and post-COVID-free matched controls over 730-day follow-up, Germany, 2021–2025

	No. of individuals		Sick leave days			Outpatient physician contacts			Outpatient ED visits		
	**Post-COVID**	**Post-COVID-free**	**Post-COVID**	**Post-COVID-free**	**Diff.**	**Post-COVID**	**Post-COVID-free**	**Diff.**	**Post-COVID**	**Post-COVID-free**	**Diff.**
**Total population**	18,313	34,838	75.0 (1.0)	39.0 (0.4)	36.0	49.1 (0.3)	37.8 (0.2)	11.3	0.5 (0.0)	0.4 (0.0)	0.1
**Age group**											
0-5 years	50	98	0.0 (0.0)	0.0 (0.0)	0.0	22.6 (1.8)	19.0 (1.5)	3.6	1.4 (0.2)	0.9 (0.1)	0.6
6-11 years	177	345	0.0 (0.0)	0.0 (0.0)	0.0	21.6 (1.2)	17.8 (1.0)	3.8	0.7 (0.1)	0.7 (0.1)	0.0
12-17 years	358	690	2.2 (0.6)	1.7 (0.3)	0.5	26.4 (1.1)	19.1 (0.8)	7.3	0.7 (0.1)	0.5 (0.0)	0.2
18-39 years	4,580	8,806	66.2 (1.7)	34.5 (0.7)	31.7	38.9 (0.5)	27.2 (0.3)	11.6	0.7 (0.0)	0.5 (0.0)	0.2
40-59 years	8,727	16,663	95.7 (1.6)	49.2 (0.7)	46.5	49.1 (0.4)	37.1 (0.3)	12.0	0.5 (0.0)	0.4 (0.0)	0.1
≥60 years	4,421	8,353	51.5 (1.9)	27.0 (0.9)	24.5	62.8 (0.7)	52.1 (0.5)	10.7	0.5 (0.0)	0.4 (0.0)	0.1
**Prevalent risk condition for severe COVID-19**											
Yes	13,990	25,091	82.7 (1.2)	44.4 (0.6)	38.3	55.5 (0.3)	44.9 (0.3)	10.6	0.6 (0.0)	0.5 (0.0)	0.1
No	4,323	9,783	50.0 (1.5)	24.7 (0.5)	25.3	28.4 (0.3)	19.1 (0.2)	9.2	0.5 (0.0)	0.3 (0.0)	0.1
**Severity of first acute diagnosed COVID-19 episode**											
Non-hospitalized	17,050	32,650	74.7 (1.0)	38.8 (0.4)	35.9	48.0 (0.3)	36.3 (0.2)	11.7	0.5 (0.0)	0.4 (0.0)	0.1
Hospitalized	1,039	1,874	75.7 (4.9)	37.5 (2.5)	38.2	65.0 (1.7)	59.2 (1.5)	5.7	0.9 (0.1)	0.8 (0.1)	0.1
Hospitalized + ICU	224	314	95.8 (13.1)	63.4 (8.9)	32.4	65.4 (4.1)	81.1 (5.7)	-15.7	0.6 (0.1)	0.6 (0.1)	0.0

Abbreviations: Diff: mean difference, ED emergency department, ICU: intensive care unit, SD: standard deviation.

Table D: Mean (SD) healthcare resource utilization and sick leave days of incident post-COVID patients and post-COVID-free matched controls over 730-day follow-up, Germany, 2021–2025 (continued)

	**No. of individuals**		**Hospitalizations**			**Hospitalizations w/o ICU admission**			**Hospitalizations w/ ICU admission**		
	**Post-COVID**	**Post-COVID-free**	**Post-COVID**	**Post-COVID-free**	**Diff.**	**Post-COVID**	**Post-COVID-free**	**Diff.**	**Post-COVID**	**Post-COVID-free**	**Diff.**
**Total population**	18,313	34,838	0.7 (0.0)	0.5 (0.0)	0.1	0.6 (0.0)	0.4 (0.0)	0.1	0.1 (0.0)	0.1 (0.0)	0.0
**Age group**											
0-5 years	50	98	0.3 (0.1)	0.2 (0.1)	0.0	0.3 (0.1)	0.2 (0.1)	0.0	0.0 (0.0)	0.0 (0.0)	0.0
6-11 years	177	345	0.4 (0.1)	0.2 (0.0)	0.1	0.3 (0.1)	0.2 (0.0)	0.1	0.0 (0.0)	0.0 (0.0)	0.0
12-17 years	358	690	0.4 (0.0)	0.3 (0.0)	0.1	0.4 (0.0)	0.2 (0.0)	0.2	0.0 (0.0)	0.0 (0.0)	0.0
18-39 years	4,580	8,806	0.5 (0.0)	0.4 (0.0)	0.1	0.5 (0.0)	0.4 (0.0)	0.1	0.0 (0.0)	0.0 (0.0)	0.0
40-59 years	8,727	16,663	0.6 (0.0)	0.5 (0.0)	0.1	0.5 (0.0)	0.4 (0.0)	0.1	0.1 (0.0)	0.1 (0.0)	0.0
≥60 years	4,421	8,353	1.0 (0.0)	0.9 (0.0)	0.1	0.8 (0.0)	0.6 (0.0)	0.1	0.2 (0.0)	0.3 (0.0)	0.0
**Prevalent risk condition for severe COVID-19**											
Yes	13,990	25,091	0.8 (0.0)	0.7 (0.0)	0.1	0.6 (0.0)	0.5 (0.0)	0.1	0.1 (0.0)	0.1 (0.0)	0.0
No	4,323	9,783	0.3 (0.0)	0.2 (0.0)	0.1	0.3 (0.0)	0.2 (0.0)	0.1	0.0 (0.0)	0.0 (0.0)	0.0
**Severity of first acute diagnosed COVID-19 episode**											
Non-hospitalized	17,050	32,650	0.6 (0.0)	0.5 (0.0)	0.1	0.5 (0.0)	0.4 (0.0)	0.1	0.1 (0.0)	0.1 (0.0)	0.0
Hospitalized	1,039	1,874	1.5 (0.1)	1.8 (0.1)	-0.3	1.1 (0.1)	1.2 (0.1)	-0.1	0.4 (0.1)	0.6 (0.1)	-0.2
Hospitalized + ICU	224	314	1.9 (0.2)	2.1 (0.2)	-0.2	0.0 (0.0)	0.0 (0.0)	0.0	1.9 (0.2)	2.1 (0.2)	-0.2

Abbreviations: Diff: mean difference, ED emergency department, ICU: intensive care unit, SD: standard deviation.

Table D: Mean (SD) healthcare resource utilization and sick leave days of incident post-COVID patients and post-COVID-free matched controls over 730-day follow-up, Germany, 2021–2025 (continued)

	No. of individuals		Hospital days			Drug prescriptions			Medical aid prescriptions		
	Post-COVID	Post-COVID-free	Post-COVID	Post-COVID-free	Diff.	Post-COVID	Post-COVID-free	Diff.	Post-COVID	Post-COVID-free	Diff.
**Total population**	18,313	34,838	5.0 (0.1)	4.1 (0.1)	0.9	27.2 (0.2)	24.1 (0.2)	3.1	2.8 (0.1)	2.5 (0.0)	0.3
**Age group**											
0-5 years	50	98	0.6 (0.3)	0.4 (0.2)	0.2	16.9 (1.8)	14.3 (1.6)	2.6	0.6 (0.2)	0.6 (0.2)	0.1
6-11 years	177	345	1.8 (0.6)	1.2 (0.4)	0.6	13.2 (1.2)	11.0 (1.3)	2.2	1.4 (0.5)	1.4 (0.4)	0.0
12-17 years	358	690	4.3 (1.3)	2.9 (0.6)	1.5	10.3 (0.6)	7.5 (0.4)	2.9	1.1 (0.1)	1.5 (0.4)	-0.4
18-39 years	4,580	8,806	3.4 (0.2)	3.1 (0.2)	0.3	13.1 (0.3)	10.1 (0.2)	3.0	1.4 (0.1)	1.1 (0.1)	0.2
40-59 years	8,727	16,663	4.6 (0.2)	3.4 (0.1)	1.1	25.4 (0.3)	22.2 (0.2)	3.2	2.5 (0.1)	2.1 (0.1)	0.4
≥60 years	4,421	8,353	7.7 (0.3)	6.9 (0.3)	0.8	47.4 (0.7)	44.2 (0.5)	3.2	5.1 (0.2)	4.9 (0.2)	0.1
**Prevalent risk condition for severe COVID**											
Yes	13,990	25,091	5.8 (0.2)	5.2 (0.1)	0.6	32.9 (0.3)	30.9 (0.2)	1.9	3.3 (0.1)	3.2 (0.1)	0.1
No	4,323	9,783	2.3 (0.2)	1.4 (0.1)	0.9	8.9 (0.2)	6.3 (0.1)	2.6	1.0 (0.0)	0.7 (0.0)	0.3
**Severity of first acute diagnosed COVID-19 episode**											
Non-hospitalized	17,050	32,650	4.4 (0.1)	3.2 (0.1)	1.2	25.4 (0.2)	21.8 (0.2)	3.6	2.4 (0.1)	2.2 (0.0)	0.3
Hospitalized	1,039	1,874	12.7 (0.9)	20.3 (1.5)	-7.7	51.4 (1.8)	58.7 (1.6)	-7.4	6.7 (0.7)	7.6 (0.5)	-0.9
Hospitalized + ICU	224	314	20.9 (3.8)	22.9 (3.6)	-2.0	69.3 (5.4)	89.4 (5.3)	-20.1	13.4 (2.4)	18.0 (2.7)	-4.7

Abbreviations: Diff: mean difference, ED emergency department, ICU: intensive care unit, SD: standard deviation.

Table E: Mean (SD) SHI-borne direct healthcare and sickness benefit costs in € of incident post-COVID patients and post-COVID-free matched controls over 365-day follow-up, Germany, 2021–2025

	**No. of individuals**		**Total costs**			**Sickness benefits**		
	**Post-COVID**	**Post-COVID-free**	**Post-COVID**	**Post-COVID-free**	**Diff.**	**Post-COVID**	**Post-COVID-free**	**Diff.**
**Total population**	23,046	43,734	5,794.7 (87.0)	3,988.5 (61.8)	1,806.2	1,459.5 (31.7)	382.6 (11.3)	1,076.9
**Age group**								
0-5 years	56	109	791.9 (133.9)	929.3 (160.4)	-137.4	0.0 (0.0)	0.0 (0.0)	0.0
6-11 years	201	392	1,718.3 (291.2)	1,212.3 (296.0)	506.1	0.0 (0.0)	0.0 (0.0)	0.0
12-17 years	424	812	2,150.9 (394.2)	1,491.8 (169.6)	659.1	0.3 (0.3)	0.1 (0.1)	0.2
18-39 years	5,655	10,857	3,983.1 (156.4)	2,394.2 (98.7)	1,588.9	1,123.7 (56.8)	209.7 (15.6)	914.0
40-59 years	11,054	21,029	5,847.2 (120.8)	3,775.5 (90.5)	2,071.7	1,870.9 (50.9)	507.3 (18.5)	1,363.6
≥60 years	5,656	10,680	7,968.6 (207.1)	6,286.3 (143.2)	1,682.3	1,149.6 (57.8)	347.5 (21.5)	802.1
**Prevalent risk condition**								
Yes	17,610	31,498	6,758.5 (105.2)	5,059.7 (83.4)	1,698.8	1,671.8 (38.5)	481.5 (14.9)	1,190.3
No	5,436	12,300	2,668.9 (132.0)	1,179.8 (37.4)	1,489.1	771.2 (48.8)	123.2 (10.9)	648.0
**Severity of first acute diagnosed COVID-19 episode**								
Non-hospitalized	21,520	41,086	5,322.8 (81.9)	3,347.7 (47.5)	1,975.1	1,422.2 (32.3)	353.2 (10.8)	1,068.9
Hospitalized	1,280	2,303	11,450.2 (567.1)	13,276.8 (708.2)	-1,826.5	1,742.8 (147.0)	679.8 (77.5)	1,063.0
Hospitalized + ICU	246	345	20,544.2 (2,468.4)	24,864.9 (2,897.6)	-4,320.7	3,482.4 (514.6)	2,239.2 (391.0)	1,243.2
Diff: mean difference								

Table E: Mean (SD) SHI-borne direct healthcare and sickness benefit costs in € of incident post-COVID patients and post-COVID-free matched controls over 365-day follow-up, Germany, 2021–2025 (continued)

	No. of individuals		Outpatient costs			Inpatient costs		
	**Post-COVID**	**Post-COVID-free**	**Post-COVID**	**Post-COVID-free**	**Diff.**	**Post-COVID**	**Post-COVID-free**	**Diff.**
**Total population**	23,046	43,734	1,358.2 (12.0)	965.7 (8.9)	392.5	1,596.2 (49.7)	1,312.9 (34.5)	283.3
**Age group**								
0-5 years	56	109	509.7 (52.9)	457.7 (49.8)	51.9	137.7 (89.7)	298.1 (111.1)	-160.3
6-11 years	201	392	664.7 (57.1)	467.2 (38.6)	197.5	747.8 (223.9)	477.3 (255.6)	270.4
12-17 years	424	812	754.7 (44.9)	510.4 (31.5)	244.3	661.3 (137.1)	604.4 (122.0)	56.8
18-39 years	5,655	10,857	1,108.4 (18.8)	718.5 (12.8)	389.9	898.1 (62.3)	846.3 (66.8)	51.8
40-59 years	11,054	21,029	1,324.7 (13.4)	927.7 (11.6)	397.0	1,404.0 (67.7)	1,013.9 (42.3)	390.1
≥60 years	5,656	10,680	1,753.4 (36.0)	1,335.1 (24.7)	418.3	2,792.3 (138.4)	2,459.7 (89.7)	332.6
**Prevalent risk condition**								
Yes	17,610	31,498	1,554.9 (15.0)	1,171.3 (12.0)	383.6	1,872.2 (60.1)	1,657.1 (46.3)	215.1
No	5,436	12,300	720.3 (10.1)	425.6 (5.0)	294.7	701.0 (79.3)	410.2 (28.1)	290.8
**Severity of first acute diagnosed COVID-19 episode**								
Non-hospitalized	21,520	41,086	1,314.4 (11.1)	912.8 (7.7)	401.7	1,328.0 (40.6)	975.8 (24.1)	352.2
Hospitalized	1,280	2,303	2,016.2 (99.6)	1,715.5 (84.3)	300.7	4,711.9 (414.1)	6,343.1 (435.4)	-1,631.2
Hospitalized + ICU	246	345	2,038.0 (247.0)	2,817.1 (401.6)	-779.1	10,487.3 (2,213.5)	11,296.0 (1,830.7)	-808.7

Diff: mean difference

Table E: Mean (SD) SHI-borne direct healthcare and sickness benefit costs in € of incident post-COVID patients and post-COVID-free matched controls over 365-day follow-up, Germany, 2021–2025 (continued)

	No. of individuals		Prescription drug costs			Prescription medical aid costs		
	**Post-COVID**	**Post-COVID-free**	**Post-COVID**	**Post-COVID-free**	**Diff.**	**Post-COVID**	**Post-COVID-free**	**Diff.**
**Total population**	23,046	43,734	1,192.7 (46.5)	1,144.0 (39.6)	48.7	188.0 (7.9)	183.2 (7.8)	4.8
**Age group**								
0-5 years	56	109	123.3 (18.9)	118.8 (18.2)	4.5	21.2 (8.4)	54.7 (40.7)	-33.5
6-11 years	201	392	236.7 (54.2)	168.9 (33.0)	67.8	69.2 (28.6)	98.8 (44.8)	-29.6
12-17 years	424	812	582.6 (352.7)	162.8 (21.4)	419.8	152.0 (81.3)	214.0 (78.6)	-62.0
18-39 years	5,655	10,857	773.9 (116.6)	536.7 (55.3)	237.2	79.0 (6.1)	82.8 (6.5)	-3.8
40-59 years	11,054	21,029	1,076.5 (57.1)	1,168.9 (66.5)	-92.4	171.1 (11.5)	157.7 (12.9)	13.3
≥60 years	5,656	10,680	1,934.0 (95.9)	1,809.3 (75.0)	124.7	339.3 (21.3)	334.7 (16.9)	4.5
**Prevalent risk condition**								
Yes	17,610	31,498	1,433.7 (58.1)	1,508.2 (54.4)	-74.5	225.9 (8.7)	241.5 (10.8)	-15.6
No	5,436	12,300	411.2 (56.7)	190.2 (14.1)	220.9	65.2 (17.9)	30.5 (1.7)	34.6
**Severity of first acute diagnosed COVID-19 episode**								
Non-hospitalized	21,520	41,086	1,103.8 (48.0)	963.4 (30.6)	140.4	154.4 (7.3)	142.5 (5.9)	11.9
Hospitalized	1,280	2,303	2,399.2 (183.0)	3,868.6 (492.9)	-1,469.5	580.2 (59.6)	669.8 (93.2)	-89.6
Hospitalized + ICU	246	345	3,242.7 (593.0)	6,278.7 (1,647.8)	-3,036.0	1,293.8 (234.0)	2,233.9 (413.8)	-940.1
Diff: mean difference								

Table F: Mean (SD) SHI-borne direct healthcare and sickness benefit costs in € of incident post-COVID patients and post-COVID-free matched controls over 730-day follow-up, Germany, 2021–2025

	No. of individuals		Total costs			Sickness benefits		
	Post-COVID	Post-COVID-free	Post-COVID	Post-COVID-free	Diff.	Post-COVID	Post-COVID-free	Diff.
**Total population**	18,313	34,838	10,362.9 (161.2)	7,928.6 (123.6)	2,434.3	2,173.9 (49.6)	763.6 (20.5)	1,410.4
**Age group**								
0-5 years	50	98	1,781.4 (300.2)	1,540.1 (230.3)	241.2	0.0 (0.0)	0.0 (0.0)	0.0
6-11 years	177	345	2,785.3 (460.2)	2,383.2 (420.7)	402.1	0.0 (0.0)	0.0 (0.0)	0.0
12-17 years	358	690	5,202.8 (1,375.3)	3,397.6 (404.3)	1,805.3	0.1 (0.1)	0.3 (0.2)	-0.1
18-39 years	4,580	8,806	6,991.9 (322.1)	4,511.2 (141.2)	2,480.6	1,601.0 (85.0)	430.4 (28.2)	1,170.5
40-59 years	8,727	16,663	10,288.6 (206.6)	7,736.6 (193.4)	2,551.9	2,841.0 (79.8)	1,022.7 (33.6)	1,818.4
≥60 years	4,421	8,353	14,786.3 (386.8)	12,428.8 (288.2)	2,357.5	1,687.2 (93.3)	663.7 (40.6)	1,023.5
**Prevalent risk condition for severe COVID**								
Yes	13,990	25,091	12,061.8 (198.1)	9,934.4 (163.8)	2,127.4	2,473.3 (60.0)	939.1 (26.7)	1,534.3
No	4,323	9,783	4,854.5 (212.8)	2,698.0 (111.2)	2,156.5	1,203.2 (77.9)	305.5 (23.3)	897.7
**Severity of first acute diagnosed COVID-19 episode**								
Non-hospitalized	17,050	32,650	9,592.7 (154.5)	6,858.4 (97.3)	2,734.4	2,125.4 (50.7)	726.5 (20.3)	1,398.9
Hospitalized	1,039	1,874	19,385.0 (958.7)	24,110.2 (1,453.5)	-4,725.2	2,570.3 (231.7)	1,151.7 (129.5)	1,418.5
Hospitalized + ICU	224	314	33,938.1 (4,005.9)	38,644.1 (4,784.4)	-4,706.0	4,466.0 (677.1)	2,911.3 (511.1)	1,554.7
Diff: mean difference, ICU: intensive care unit								

Table F: Mean (SD) SHI-borne direct healthcare and sickness benefit costs in € of incident post-COVID patients and post-COVID-free matched controls over 730-day follow-up, Germany, 2021–2025 (continued)

	No. of individuals		Outpatient costs			Inpatient costs		
	**Post-COVID**	**Post-COVID-free**	**Post-COVID**	**Post-COVID-free**	**Diff.**	**Post-COVID**	**Post-COVID-free**	**Diff.**
**Total population**	18,313	34,838	2,599.5 (26.9)	1,939.7 (18.9)	659.8	2,828.5 (77.1)	2,546.6 (60.6)	281.9
**Age group**								
0-5 years	50	98	1,011.7 (98.0)	849.7 (79.6)	162.0	502.5 (240.5)	380.8 (140.0)	121.7
6-11 years	177	345	1,188.4 (95.9)	929.6 (75.6)	258.8	1,012.1 (286.8)	906.2 (281.1)	105.9
12-17 years	358	690	1,318.3 (80.5)	1,036.4 (65.1)	282.0	2,030.9 (626.3)	1,641.1 (323.7)	389.8
18-39 years	4,580	8,806	2,065.0 (40.2)	1,437.4 (28.4)	627.6	1,576.6 (95.8)	1,527.3 (99.3)	49.3
40-59 years	8,727	16,663	2,547.1 (28.4)	1,879.4 (24.8)	667.7	2,447.7 (102.1)	2,068.9 (76.1)	378.8
≥60 years	4,421	8,353	3,432.2 (85.4)	2,686.5 (51.5)	745.6	5,051.2 (217.3)	4,727.1 (167.6)	324.1
**Prevalent risk condition for severe COVID**								
Yes	13,990	25,091	2,971.7 (33.9)	2,333.3 (25.3)	638.4	3,310.2 (95.7)	3,162.3 (76.6)	147.9
No	4,323	9,783	1,392.7 (21.3)	911.8 (11.3)	480.9	1,266.8 (99.2)	940.7 (86.2)	326.1
**Severity of first acute diagnosed COVID-19 episode**								
Non-hospitalized	17,050	32,650	2,517.0 (24.8)	1,838.1 (16.3)	678.8	2,421.5 (65.5)	2,029.4 (49.1)	392.0
Hospitalized	1,039	1,874	3,733.0 (220.7)	3,402.1 (182.0)	330.9	7,535.4 (599.1)	10,674.8 (676.4)	-3,139.4
Hospitalized + ICU	224	314	4,344.0 (635.2)	5,312.1 (780.3)	-968.1	15,577.3 (2,949.1)	15,447.6 (2,378.0)	129.7
Diff: mean difference, ICU: intensive care unit								

Table F: Mean (SD) SHI-borne direct healthcare and sickness benefit costs in € of incident post-COVID patients and post-COVID-free matched controls over 730-day follow-up, Germany, 2021–2025 (continued)

	No. of individuals		Prescription drug costs			Prescription medical aid costs		
	**Post-COVID**	**Post-COVID-free**	**Post-COVID**	**Post-COVID-free**	**Diff.**	**Post-COVID**	**Post-COVID-free**	**Diff.**
**Total population**	18,313	34,838	2,396.0 (103.5)	2,328.8 (85.2)	67.2	365.0 (11.6)	349.9 (14.4)	15.1
**Age group**								
0-5 years	50	98	236.8 (34.3)	245.4 (44.0)	-8.6	30.4 (10.7)	64.3 (47.3)	-33.9
6-11 years	177	345	432.9 (107.0)	316.7 (64.5)	116.3	151.9 (72.2)	230.8 (106.0)	-78.9
12-17 years	358	690	1,736.0 (1,220.9)	350.4 (55.3)	1,385.6	117.4 (40.3)	369.4 (153.1)	-252.0
18-39 years	4,580	8,806	1,586.2 (271.8)	956.8 (56.3)	629.4	163.0 (12.1)	159.2 (11.6)	3.8
40-59 years	8,727	16,663	2,116.6 (118.3)	2,448.9 (150.2)	-332.4	336.2 (16.3)	316.8 (25.3)	19.4
≥60 years	4,421	8,353	3,953.1 (206.3)	3,734.7 (168.2)	218.4	662.7 (32.7)	616.8 (24.6)	45.9
**Prevalent risk condition for severe COVID**								
Yes	13,990	25,091	2,859.8 (127.0)	3,040.4 (116.7)	-180.7	446.8 (15.0)	459.3 (19.9)	-12.5
No	4,323	9,783	892.2 (149.8)	475.0 (37.6)	417.2	99.6 (7.2)	65.0 (2.6)	34.6
**Severity of first acute diagnosed COVID-19 episode**								
Non-hospitalized	17,050	32,650	2,223.7 (106.3)	1,974.3 (61.5)	249.4	305.2 (9.9)	290.0 (13.7)	15.2
Hospitalized	1,039	1,874	4,475.4 (385.5)	7,835.2 (1,170.9)	-3,359.8	1,071.0 (106.3)	1,046.4 (75.3)	24.7
Hospitalized + ICU	224	314	7,386.3 (1,708.4)	11,580.3 (3,375.6)	-4,194.0	2,164.4 (361.7)	3,392.8 (652.3)	-1,228.4
Diff: mean difference, ICU: intensive care unit								

Table G: Mean (SD) healthcare resource utilization and sick leave days of incident post-COVID patients and post-COVID-free matched controls over 365-day follow-up, Germany, 2021–2025, sensitivity analyses with newly-occurring diagnosed post-COVID-related health outcomes as censoring events

	No. of individuals		Sick leave days			Outpatient physician contacts			Outpatient ED visits		
	**Post-COVID**	**Post-COVID-free**	**Post-COVID**	**Post-COVID-free**	**Diff.**	**Post-COVID**	**Post-COVID-free**	**Diff.**	**Post-COVID**	**Post-COVID-free**	**Diff.**
**Total population**	23,046	43,734	46.1 (0.6)	12.9 (0.3)	33.2	25.8 (0.1)	14.6 (0.1)	11.2	0.3 (0.0)	0.1 (0.0)	0.2
**Age group**											
0-5 years	56	109	0.0 (0.0)	0.0 (0.0)	0.0	12.4 (1.1)	8.3 (0.7)	4.1	0.8 (0.2)	0.3 (0.1)	0.6
6-11 years	201	392	0.0 (0.0)	0.0 (0.0)	0.0	12.3 (0.7)	7.5 (0.6)	4.8	0.5 (0.1)	0.2 (0.0)	0.3
12-17 years	424	812	0.9 (0.2)	0.3 (0.1)	0.7	14.8 (0.6)	7.2 (0.3)	7.6	0.4 (0.0)	0.2 (0.0)	0.2
18-39 years	5,655	10,857	40.5 (1.0)	11.2 (0.4)	29.2	21.0 (0.2)	10.2 (0.2)	10.9	0.4 (0.0)	0.2 (0.0)	0.2
40-59 years	11,054	21,029	58.0 (0.9)	16.0 (0.4)	42.0	25.7 (0.2)	14.1 (0.2)	11.5	0.2 (0.0)	0.1 (0.0)	0.1
≥60 years	5,656	10,680	33.4 (1.1)	9.9 (0.5)	23.6	32.2 (0.3)	21.2 (0.3)	11.0	0.3 (0.0)	0.1 (0.0)	0.1
**Prevalent risk condition for severe COVID**											
Yes	17,610	31,498	51.2 (0.7)	15.3 (0.4)	35.9	29.1 (0.2)	18.3 (0.2)	10.8	0.3 (0.0)	0.1 (0.0)	0.2
No	5,436	12,300	29.7 (0.9)	7.7 (0.3)	22.0	15.1 (0.1)	6.8 (0.1)	8.3	0.2 (0.0)	0.1 (0.0)	0.1
**Severity of first acute diagnosed COVID-19 episode**											
Non-hospitalized	21,520	41,086	45.8 (0.6)	12.6 (0.3)	33.2	25.2 (0.1)	14.0 (0.1)	11.2	0.3 (0.0)	0.1 (0.0)	0.2
Hospitalized	1,280	2,303	47.7 (3.0)	15.8 (2.1)	31.9	34.5 (0.8)	24.2 (0.8)	10.3	0.5 (0.0)	0.3 (0.0)	0.2
Hospitalized + ICU	246	345	66.4 (8.3)	33.8 (9.0)	32.6	34.4 (1.7)	42.4 (6.7)	-8.0	0.3 (0.1)	0.2 (0.1)	0.1

Abbreviations: Diff: mean difference, ED emergency department, ICU: intensive care unit, SD: standard deviation.

Table G: Mean (SD) healthcare resource utilization and sick leave days of incident post-COVID patients and post-COVID-free matched controls over 365-day follow-up, Germany, 2021–2025, sensitivity analyses with newly-occurring diagnosed post-COVID-related health outcomes as censoring events (continued)

	No. of individuals		Hospitalizations			Hospitalizations w/o ICU admission			Hospitalizations w/ ICU admission		
	Post-COVID	Post-COVID-free	Post-COVID	Post-COVID-free	Diff.	Post-COVID	Post-COVID-free	Diff.	Post-COVID	Post-COVID-free	Diff.
**Total population**	23,046	43,734	0.4 (0.0)	0.1 (0.0)	0.2	0.3 (0.0)	0.1 (0.0)	0.2	0.1 (0.0)	0.0 (0.0)	0.0
**Age group**											
0-5 years	56	109	0.1 (0.1)	0.0 (0.0)	0.1	0.1 (0.1)	0.0 (0.0)	0.1	0.0 (0.0)	0.0 (0.0)	0.0
6-11 years	201	392	0.2 (0.0)	0.0 (0.0)	0.2	0.2 (0.0)	0.0 (0.0)	0.2	0.0 (0.0)	0.0 (0.0)	0.0
12-17 years	424	812	0.2 (0.0)	0.1 (0.0)	0.1	0.2 (0.0)	0.1 (0.0)	0.1	0.0 (0.0)	0.0 (0.0)	0.0
18-39 years	5,655	10,857	0.3 (0.0)	0.1 (0.0)	0.1	0.3 (0.0)	0.1 (0.0)	0.1	0.0 (0.0)	0.0 (0.0)	0.0
40-59 years	11,054	21,029	0.3 (0.0)	0.1 (0.0)	0.2	0.3 (0.0)	0.1 (0.0)	0.2	0.0 (0.0)	0.0 (0.0)	0.0
≥60 years	5,656	10,680	0.6 (0.0)	0.2 (0.0)	0.3	0.4 (0.0)	0.2 (0.0)	0.3	0.1 (0.0)	0.1 (0.0)	0.1
**Prevalent risk condition for severe COVID**											
Yes	17,610	31,498	0.4 (0.0)	0.2 (0.0)	0.2	0.4 (0.0)	0.1 (0.0)	0.2	0.1 (0.0)	0.0 (0.0)	0.0
No	5,436	12,300	0.2 (0.0)	0.1 (0.0)	0.1	0.2 (0.0)	0.1 (0.0)	0.1	0.0 (0.0)	0.0 (0.0)	0.0
**Severity of first acute diagnosed COVID-19 episode**											
Non-hospitalized	21,520	41,086	0.3 (0.0)	0.1 (0.0)	0.2	0.3 (0.0)	0.1 (0.0)	0.2	0.0 (0.0)	0.0 (0.0)	0.0
Hospitalized	1,280	2,303	0.9 (0.0)	0.6 (0.0)	0.3	0.7 (0.0)	0.4 (0.0)	0.3	0.3 (0.0)	0.2 (0.0)	0.0
Hospitalized + ICU	246	345	1.1 (0.1)	0.6 (0.1)	0.5	0.0 (0.0)	0.0 (0.0)	0.0	1.1 (0.1)	0.6 (0.1)	0.5

Abbreviations: Diff: mean difference, ED emergency department, ICU: intensive care unit, SD: standard deviation.

Table G: Mean (SD) healthcare resource utilization and sick leave days of incident post-COVID patients and post-COVID-free matched controls over 365-day follow-up, Germany, 2021–2025, sensitivity analyses with newly-occurring diagnosed post-COVID-related health outcomes as censoring events (continued)

	No. of individuals		Hospital days			Drug prescriptions			Medical aid prescriptions		
	**Post-COVID**	**Post-COVID-free**	**Post-COVID**	**Post-COVID-free**	**Diff.**	**Post-COVID**	**Post-COVID-free**	**Diff.**	**Post-COVID**	**Post-COVID-free**	**Diff.**
**Total population**	23,046	43,734	2.8 (0.1)	1.2 (0.1)	1.6	13.8 (0.1)	9.7 (0.1)	4.1	1.4 (0.0)	1.0 (0.0)	0.4
**Age group**											
0-5 years	56	109	0.2 (0.1)	0.1 (0.1)	0.1	8.7 (1.0)	6.4 (0.9)	2.3	0.4 (0.1)	0.3 (0.1)	0.1
6-11 years	201	392	1.3 (0.5)	0.1 (0.1)	1.2	7.7 (0.6)	4.5 (0.5)	3.2	0.7 (0.2)	0.6 (0.2)	0.1
12-17 years	424	812	1.4 (0.4)	0.8 (0.2)	0.6	5.4 (0.3)	3.1 (0.2)	2.3	0.8 (0.2)	0.8 (0.2)	0.0
18-39 years	5,655	10,857	2.0 (0.1)	1.2 (0.1)	0.8	6.9 (0.1)	3.8 (0.1)	3.1	0.6 (0.0)	0.5 (0.0)	0.2
40-59 years	11,054	21,029	2.6 (0.1)	1.1 (0.1)	1.6	12.7 (0.1)	8.7 (0.1)	4.0	1.2 (0.0)	0.8 (0.0)	0.4
≥60 years	5,656	10,680	4.3 (0.2)	1.6 (0.2)	2.7	23.7 (0.3)	18.6 (0.3)	5.1	2.6 (0.1)	2.0 (0.1)	0.6
**Prevalent risk condition for severe COVID**											
Yes	17,610	31,498	3.3 (0.1)	1.6 (0.1)	1.7	16.6 (0.1)	13.3 (0.1)	3.4	1.7 (0.0)	1.4 (0.0)	0.3
No	5,436	12,300	1.2 (0.1)	0.4 (0.1)	0.8	4.6 (0.1)	2.2 (0.0)	2.4	0.5 (0.0)	0.3 (0.0)	0.2
**Severity of first acute diagnosed COVID-19 episode**											
Non-hospitalized	21,520	41,086	2.4 (0.1)	0.9 (0.0)	1.6	12.8 (0.1)	8.8 (0.1)	4.0	1.2 (0.0)	0.9 (0.0)	0.3
Hospitalized	1,280	2,303	7.7 (0.6)	7.7 (1.0)	0.0	28.0 (0.8)	25.9 (1.0)	2.1	3.7 (0.3)	3.3 (0.3)	0.5
Hospitalized + ICU	246	345	14.2 (2.5)	8.5 (3.0)	5.8	35.6 (2.5)	40.0 (3.4)	-4.4	7.4 (1.2)	6.4 (1.1)	1.0
Abbreviations: Diff: mean difference, ED emergency department, ICU: intensive care unit, SD: standard deviation.											

Table H: Mean (SD) healthcare resource utilization and sick leave days of incident post-COVID patients and post-COVID-free matched controls over 730-day follow-up, Germany, 2021–2025, sensitivity analyses with newly-occurring diagnosed post-COVID-related health outcomes as censoring events

	**No. of individuals**		**Sick leave days**			**Outpatient physician contacts**			**Outpatient ED visits**		
	**Post-COVID**	**Post-COVID-free**	**Post-COVID**	**Post-COVID-free**	**Diff.**	**Post-COVID**	**Post-COVID-free**	**Diff.**	**Post-COVID**	**Post-COVID-free**	**Diff.**
**Total population**	18,313	34,838	75.0 (1.0)	21.0 (0.5)	54.0	49.1 (0.3)	26.6 (0.3)	22.5	0.5 (0.0)	0.2 (0.0)	0.3
**Age group**											
0-5 years	50	98	0.0 (0.0)	0.0 (0.0)	0.0	22.6 (1.8)	14.1 (1.2)	8.5	1.4 (0.2)	0.5 (0.2)	0.9
6-11 years	177	345	0.0 (0.0)	0.0 (0.0)	0.0	21.6 (1.2)	13.4 (1.4)	8.2	0.7 (0.1)	0.5 (0.1)	0.3
12-17 years	358	690	2.2 (0.6)	0.5 (0.3)	1.7	26.4 (1.1)	13.4 (0.8)	13.0	0.7 (0.1)	0.3 (0.0)	0.4
18-39 years	4,580	8,806	66.2 (1.7)	19.2 (0.9)	47.0	38.9 (0.5)	17.9 (0.5)	20.9	0.7 (0.0)	0.3 (0.0)	0.5
40-59 years	8,727	16,663	95.7 (1.6)	27.0 (0.9)	68.7	49.1 (0.4)	26.3 (0.5)	22.8	0.5 (0.0)	0.2 (0.0)	0.3
≥60 years	4,421	8,353	51.5 (1.9)	14.2 (1.0)	37.3	62.8 (0.7)	38.9 (0.7)	23.9	0.5 (0.0)	0.2 (0.0)	0.3
**Prevalent risk condition for severe COVID**											
Yes	13,990	25,091	82.7 (1.2)	25.0 (0.8)	57.8	55.5 (0.3)	33.9 (0.5)	21.5	0.6 (0.0)	0.2 (0.0)	0.3
No	4,323	9,783	50.0 (1.5)	13.6 (0.6)	36.4	28.4 (0.3)	12.9 (0.2)	15.5	0.5 (0.0)	0.2 (0.0)	0.3
**Severity of first acute diagnosed COVID-19 episode**											
Non-hospitalized	17,050	32,650	74.7 (1.0)	21.0 (0.6)	53.7	48.0 (0.3)	25.7 (0.3)	22.3	0.5 (0.0)	0.2 (0.0)	0.3
Hospitalized	1,039	1,874	75.7 (4.9)	18.6 (3.1)	57.1	65.0 (1.7)	42.7 (2.0)	22.2	0.9 (0.1)	0.4 (0.0)	0.5
Hospitalized + ICU	224	314	95.8 (13.1)	39.3 (12.8)	56.6	65.4 (4.1)	75.5 (17.1)	-10.0	0.6 (0.1)	0.3 (0.1)	0.3
Abbreviations: Diff: mean difference, ED emergency department, ICU: intensive care unit, SD: standard deviation.											

Table H: Mean (SD) healthcare resource utilization and sick leave days of incident post-COVID patients and post-COVID-free matched controls over 730-day follow-up, Germany, 2021–2025, sensitivity analyses with newly-occurring diagnosed post-COVID-related health outcomes as censoring events (continued)

	No. of individuals		Hospitalizations			Hospitalizations w/o ICU admission			Hospitalizations w/ ICU admission		
	Post-COVID	Post-COVID-free	Post-COVID	Post-COVID-free	Diff.	Post-COVID	Post-COVID-free	Diff.	Post-COVID	Post-COVID-free	Diff.
**Total population**	18,313	34,838	0.7 (0.0)	0.2 (0.0)	0.4	0.6 (0.0)	0.2 (0.0)	0.4	0.1 (0.0)	0.0 (0.0)	0.1
**Age group**											
0-5 years	50	98	0.3 (0.1)	0.1 (0.0)	0.2	0.3 (0.1)	0.1 (0.0)	0.2	0.0 (0.0)	0.0 (0.0)	0.0
6-11 years	177	345	0.4 (0.1)	0.1 (0.0)	0.2	0.3 (0.1)	0.1 (0.0)	0.3	0.0 (0.0)	0.0 (0.0)	0.0
12-17 years	358	690	0.4 (0.0)	0.2 (0.0)	0.2	0.4 (0.0)	0.1 (0.0)	0.3	0.0 (0.0)	0.0 (0.0)	0.0
18-39 years	4,580	8,806	0.5 (0.0)	0.2 (0.0)	0.3	0.5 (0.0)	0.2 (0.0)	0.3	0.0 (0.0)	0.0 (0.0)	0.0
40-59 years	8,727	16,663	0.6 (0.0)	0.2 (0.0)	0.4	0.5 (0.0)	0.2 (0.0)	0.3	0.1 (0.0)	0.0 (0.0)	0.0
≥60 years	4,421	8,353	1.0 (0.0)	0.3 (0.0)	0.7	0.8 (0.0)	0.3 (0.0)	0.5	0.2 (0.0)	0.1 (0.0)	0.2
**Prevalent risk condition for severe COVID**											
Yes	13,990	25,091	0.8 (0.0)	0.3 (0.0)	0.5	0.6 (0.0)	0.2 (0.0)	0.4	0.1 (0.0)	0.1 (0.0)	0.1
No	4,323	9,783	0.3 (0.0)	0.1 (0.0)	0.2	0.3 (0.0)	0.1 (0.0)	0.2	0.0 (0.0)	0.0 (0.0)	0.0
**Severity of first acute diagnosed COVID-19 episode**											
Non-hospitalized	17,050	32,650	0.6 (0.0)	0.2 (0.0)	0.4	0.5 (0.0)	0.2 (0.0)	0.4	0.1 (0.0)	0.0 (0.0)	0.0
Hospitalized	1,039	1,874	1.5 (0.1)	0.9 (0.1)	0.6	1.1 (0.1)	0.7 (0.1)	0.4	0.4 (0.1)	0.2 (0.1)	0.2
Hospitalized + ICU	224	314	1.9 (0.2)	1.0 (0.2)	0.9	0.0 (0.0)	0.0 (0.0)	0.0	1.9 (0.2)	1.0 (0.2)	0.9

Abbreviations: Diff: mean difference, ED emergency department, ICU: intensive care unit, SD: standard deviation.

Table H: Mean (SD) healthcare resource utilization and sick leave days of incident post-COVID patients and post-COVID-free matched controls over 730-day follow-up, Germany, 2021–2025, sensitivity analyses with newly-occurring diagnosed post-COVID-related health outcomes as censoring events (continued)

	No. of individuals		Hospital days			Drug prescriptions			Medical aid prescriptions		
	**Post-COVID**	**Post-COVID-free**	**Post-COVID**	**Post-COVID-free**	**Diff.**	**Post-COVID**	**Post-COVID-free**	**Diff.**	**Post-COVID**	**Post-COVID-free**	**Diff.**
**Total population**	18,313	34,838	5.0 (0.1)	1.9 (0.1)	3.1	27.2 (0.2)	18.0 (0.3)	9.2	2.8 (0.1)	1.8 (0.1)	0.9
**Age group**											
0-5 years	50	98	0.6 (0.3)	0.1 (0.1)	0.5	16.9 (1.8)	9.9 (1.4)	7.0	0.6 (0.2)	0.3 (0.1)	0.3
6-11 years	177	345	1.8 (0.6)	0.3 (0.1)	1.5	13.2 (1.2)	6.9 (1.1)	6.4	1.4 (0.5)	1.9 (1.0)	-0.5
12-17 years	358	690	4.3 (1.3)	3.0 (1.3)	1.4	10.3 (0.6)	6.0 (0.7)	4.4	1.1 (0.1)	1.5 (0.5)	-0.4
18-39 years	4,580	8,806	3.4 (0.2)	1.5 (0.2)	1.9	13.1 (0.3)	6.7 (0.2)	6.4	1.4 (0.1)	0.9 (0.1)	0.5
40-59 years	8,727	16,663	4.6 (0.2)	1.8 (0.2)	2.8	25.4 (0.3)	16.5 (0.4)	8.9	2.5 (0.1)	1.6 (0.1)	0.9
≥60 years	4,421	8,353	7.7 (0.3)	2.3 (0.3)	5.4	47.4 (0.7)	35.2 (0.8)	12.3	5.1 (0.2)	3.5 (0.3)	1.6
**Prevalent risk condition for severe COVID**											
Yes	13,990	25,091	5.8 (0.2)	2.4 (0.2)	3.5	32.9 (0.3)	25.4 (0.4)	7.5	3.3 (0.1)	2.6 (0.1)	0.8
No	4,323	9,783	2.3 (0.2)	0.9 (0.2)	1.4	8.9 (0.2)	4.1 (0.1)	4.8	1.0 (0.0)	0.5 (0.0)	0.5
**Severity of first acute diagnosed COVID-19 episode**											
Non-hospitalized	17,050	32,650	4.4 (0.1)	1.4 (0.1)	3.0	25.4 (0.2)	16.5 (0.3)	8.9	2.4 (0.1)	1.6 (0.1)	0.8
Hospitalized	1,039	1,874	12.7 (0.9)	11.4 (2.5)	1.3	51.4 (1.8)	46.2 (2.9)	5.2	6.7 (0.7)	5.6 (0.8)	1.1
Hospitalized + ICU	224	314	20.9 (3.8)	18.2 (10.6)	2.7	69.3 (5.4)	76.0 (8.9)	-6.7	13.4 (2.4)	15.3 (3.7)	-1.9

Abbreviations: Diff: mean difference, ED emergency department, ICU: intensive care unit, SD: standard deviation.

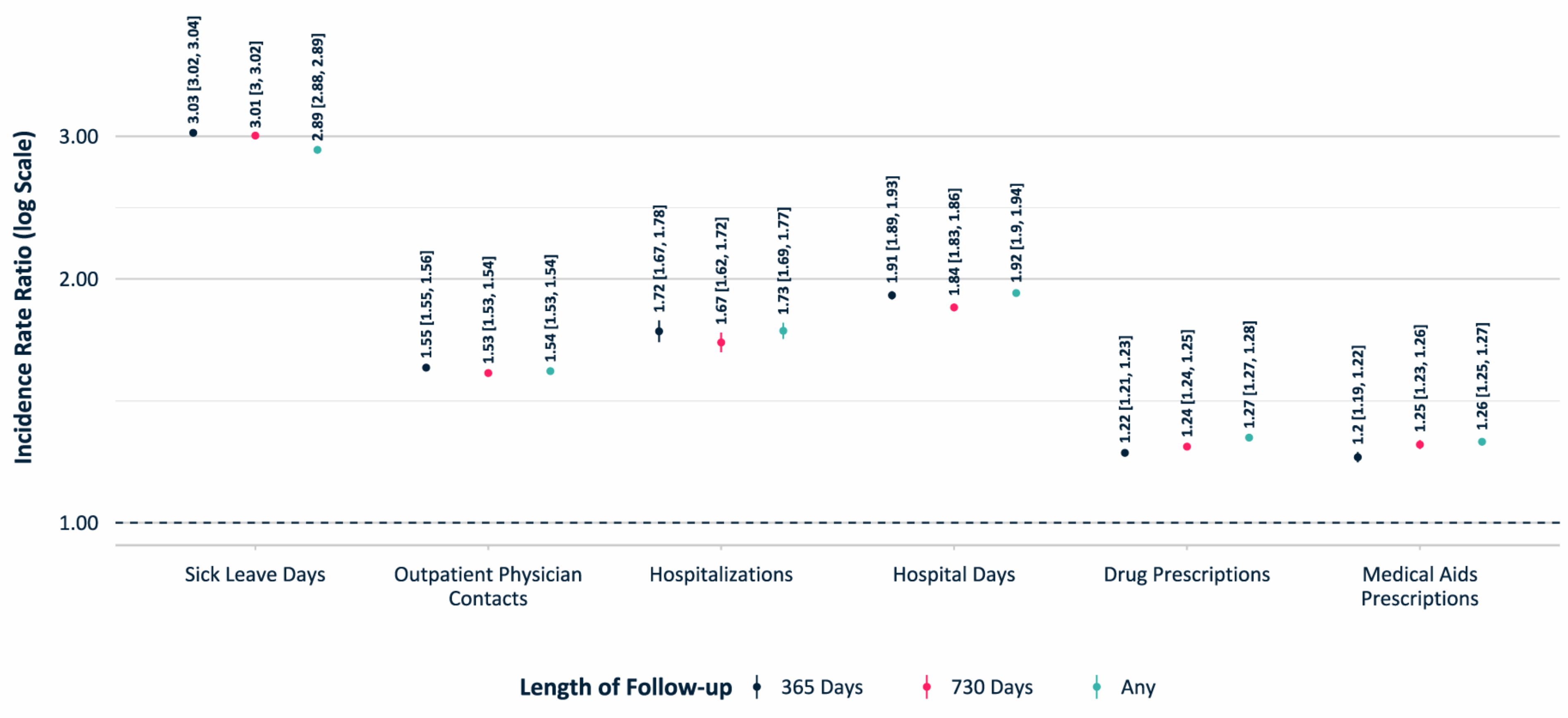

Figure A: Relative healthcare resource utilization and sick leave rates of post-COVID compared to post-COVID-free controls over 365 days, 730 days, and any follow-up period, Germany, 2021–2025, sensitivity analyses with newly-occurring diagnosed post-COVID-related health outcomes as censoring event. 95% confidence intervals given in brackets.

Table I: Mean (SD) SHI-borne direct healthcare and sickness benefit costs in € of incident post-COVID patients and post-COVID-free matched controls over 365-day follow-up, Germany, 2021–2025, sensitivity analyses with newly-occurring diagnosed post-COVID-related health outcomes as censoring event

	**No. of individuals**		**Total costs**			**Sickness benefits**		
	**Post-COVID**	**Post-COVID-free**	**Post-COVID**	**Post-COVID-free**	**Diff.**	**Post-COVID**	**Post-COVID-free**	**Diff.**
**Total population**	23,046	43,734	5,794.7 (87.0)	2,731.3 (75.0)	3,063.4	1,459.5 (31.7)	236.3 (12.8)	1,223.2
**Age group**								
0-5 years	56	109	791.9 (133.9)	555.2 (82.4)	236.8	0.0 (0.0)	0.0 (0.0)	0.0
6-11 years	201	392	1,718.3 (291.2)	803.1 (168.2)	915.3	0.0 (0.0)	0.0 (0.0)	0.0
12-17 years	424	812	2,150.9 (394.2)	1,293.6 (226.8)	857.3	0.3 (0.3)	0.2 (0.2)	0.1
18-39 years	5,655	10,857	3,983.1 (156.4)	1,789.9 (123.0)	2,193.2	1,123.7 (56.8)	140.2 (19.3)	983.6
40-59 years	11,054	21,029	5,847.2 (120.8)	2,699.9 (126.4)	3,147.3	1,870.9 (50.9)	307.5 (20.7)	1,563.4
≥60 years	5,656	10,680	7,968.6 (207.1)	3,999.5 (127.9)	3,969.1	1,149.6 (57.8)	220.5 (25.2)	929.2
**Prevalent risk condition for severe COVID**								
Yes	17,610	31,498	6,758.5 (105.2)	3,668.3 (109.0)	3,090.2	1,671.8 (38.5)	312.9 (18.1)	1,358.8
No	5,436	12,300	2,668.9 (132.0)	769.7 (31.4)	1,899.3	771.2 (48.8)	75.8 (11.5)	695.4
**Severity of first acute diagnosed COVID-19 episode**								
Non-hospitalized	21,520	41,086	5,322.8 (81.9)	2,347.2 (56.6)	2,975.6	1,422.2 (32.3)	214.6 (12.0)	1,207.5
Hospitalized	1,280	2,303	11,450.2 (567.1)	9,761.4 (1,150.9)	1,688.8	1,742.8 (147.0)	518.4 (111.7)	1,224.4
Hospitalized + ICU	246	345	20,544.2 (2,468.4)	14,027.0 (1,808.7)	6,517.2	3,482.4 (514.6)	1,805.4 (561.4)	1,677.0
Diff: mean difference, ICU: intensive care unit								

Table I: Mean (SD) SHI-borne direct healthcare and sickness benefit costs in € of incident post-COVID patients and post-COVID-free matched controls over 365-day follow-up, Germany, 2021–2025, sensitivity analyses with newly-occurring diagnosed post-COVID-related health outcomes as censoring event (continued)

	No. of individuals		Outpatient costs			Inpatient costs		
	Post-COVID	Post-COVID-free	Post-COVID	Post-COVID-free	Diff.	Post-COVID	Post-COVID-free	Diff.
**Total population**	23,046	43,734	1,358.2 (12.0)	742.5 (11.8)	615.7	1,596.2 (49.7)	628.5 (25.3)	967.6
**Age group**								
0-5 years	56	109	509.7 (52.9)	377.9 (34.3)	131.7	137.7 (89.7)	72.5 (54.4)	65.2
6-11 years	201	392	664.7 (57.1)	432.6 (50.8)	232.1	747.8 (223.9)	127.4 (74.5)	620.4
12-17 years	424	812	754.7 (44.9)	374.8 (27.7)	379.9	661.3 (137.1)	490.0 (150.1)	171.3
18-39 years	5,655	10,857	1,108.4 (18.8)	547.3 (17.8)	561.1	898.1 (62.3)	529.7 (48.8)	368.4
40-59 years	11,054	21,029	1,324.7 (13.4)	707.2 (16.7)	617.5	1,404.0 (67.7)	514.3 (33.9)	889.6
≥60 years	5,656	10,680	1,753.4 (36.0)	1,063.6 (30.7)	689.8	2,792.3 (138.4)	1,003.2 (61.1)	1,789.1
**Prevalent risk condition for severe COVID**								
Yes	17,610	31,498	1,554.9 (15.0)	946.9 (17.0)	608.0	1,872.2 (60.1)	828.2 (36.1)	1,044.0
No	5,436	12,300	720.3 (10.1)	314.1 (5.4)	406.2	701.0 (79.3)	210.5 (19.6)	490.6
**Severity of first acute diagnosed COVID-19 episode**								
Non-hospitalized	21,520	41,086	1,314.4 (11.1)	705.2 (10.7)	609.2	1,328.0 (40.6)	478.6 (18.6)	849.5
Hospitalized	1,280	2,303	2,016.2 (99.6)	1,308.4 (92.7)	707.7	4,711.9 (414.1)	3,513.0 (386.8)	1,199.0
Hospitalized + ICU	246	345	2,038.0 (247.0)	2,782.9 (789.1)	-744.9	10,487.3 (2,213.5)	3,941.1 (955.7)	6,546.2

Diff: mean difference, ICU: intensive care unit

Table I: Mean (SD) SHI-borne direct healthcare and sickness benefit costs in € of incident post-COVID patients and post-COVID-free matched controls over 365-day follow-up, Germany, 2021–2025, sensitivity analyses with newly-occurring diagnosed post-COVID-related health outcomes as censoring event (continued)

	No. of individuals		Prescription drug costs			Prescription medical aid costs		
	Post-COVID	Post-COVID-free	Post-COVID	Post-COVID-free	Diff.	Post-COVID	Post-COVID-free	Diff.
**Total population**	23,046	43,734	1,192.7 (46.5)	979.1 (62.2)	213.7	188.0 (7.9)	144.9 (9.9)	43.1
**Age group**								
0-5 years	56	109	123.3 (18.9)	87.3 (14.5)	36.0	21.2 (8.4)	17.4 (7.3)	3.8
6-11 years	201	392	236.7 (54.2)	143.9 (33.8)	92.8	69.2 (28.6)	99.1 (67.3)	-29.9
12-17 years	424	812	582.6 (352.7)	166.7 (32.4)	415.9	152.0 (81.3)	261.9 (115.1)	-109.9
18-39 years	5,655	10,857	773.9 (116.6)	488.6 (100.0)	285.3	79.0 (6.1)	84.1 (11.2)	-5.1
40-59 years	11,054	21,029	1,076.5 (57.1)	1,049.6 (111.1)	26.8	171.1 (11.5)	121.2 (17.3)	49.9
≥60 years	5,656	10,680	1,934.0 (95.9)	1,464.5 (82.7)	469.5	339.3 (21.3)	247.8 (15.4)	91.5
**Prevalent risk condition for severe COVID**								
Yes	17,610	31,498	1,433.7 (58.1)	1,377.6 (91.4)	56.1	225.9 (8.7)	202.7 (14.6)	23.2
No	5,436	12,300	411.2 (56.7)	145.3 (16.4)	265.9	65.2 (17.9)	24.0 (2.1)	41.2
**Severity of first acute diagnosed COVID-19 episode**								
Non-hospitalized	21,520	41,086	1,103.8 (48.0)	822.8 (44.1)	281.0	154.4 (7.3)	125.9 (9.9)	28.5
Hospitalized	1,280	2,303	2,399.2 (183.0)	4,003.2 (1,046.5)	-1,604.0	580.2 (59.6)	418.5 (60.5)	161.7
Hospitalized + ICU	246	345	3,242.7 (593.0)	4,193.2 (958.0)	-950.5	1,293.8 (234.0)	1,304.4 (297.6)	-10.6

Diff: mean difference, ICU: intensive care unit

Table J: Mean (SD) SHI-borne direct healthcare and sickness benefit costs in € of incident post-COVID patients and post-COVID-free matched controls over 730-day follow-up, Germany, 2021–2025, sensitivity analyses with newly-occurring diagnosed post-COVID-related health outcomes as censoring event

	**No. of individuals**		**Total costs**			**Sickness benefits**		
	**Post-COVID**	**Post-COVID-free**	**Post-COVID**	**Post-COVID-free**	**Diff.**	**Post-COVID**	**Post-COVID-free**	**Diff.**
**Total population**	18,313	34,838	10,362.9 (161.2)	4,966.7 (193.3)	5,396.2	2,173.9 (49.6)	350.7 (24.4)	1,823.2
**Age group**								
0-5 years	50	98	1,781.4 (300.2)	926.6 (155.5)	854.7	0.0 (0.0)	0.0 (0.0)	0.0
6-11 years	177	345	2,785.3 (460.2)	1,718.3 (480.7)	1,067.0	0.0 (0.0)	0.0 (0.0)	0.0
12-17 years	358	690	5,202.8 (1,375.3)	2,882.8 (704.2)	2,320.0	0.1 (0.1)	0.0 (0.0)	0.1
18-39 years	4,580	8,806	6,991.9 (322.1)	2,799.5 (172.8)	4,192.3	1,601.0 (85.0)	197.7 (34.3)	1,403.3
40-59 years	8,727	16,663	10,288.6 (206.6)	5,273.5 (346.8)	5,015.1	2,841.0 (79.8)	477.5 (40.8)	2,363.5
≥60 years	4,421	8,353	14,786.3 (386.8)	7,086.0 (364.7)	7,700.4	1,687.2 (93.3)	312.6 (50.4)	1,374.6
**Prevalent risk condition for severe COVID**								
Yes	13,990	25,091	12,061.8 (198.1)	6,762.7 (288.0)	5,299.1	2,473.3 (60.0)	468.7 (35.4)	2,004.7
No	4,323	9,783	4,854.5 (212.8)	1,579.2 (92.8)	3,275.3	1,203.2 (77.9)	128.7 (21.9)	1,074.5
**Severity of first acute diagnosed COVID-19 episode**								
Non-hospitalized	17,050	32,650	9,592.7 (154.5)	4,328.5 (149.6)	5,264.3	2,125.4 (50.7)	337.4 (24.7)	1,787.9
Hospitalized	1,039	1,874	19,385.0 (958.7)	17,709.3 (3,064.1)	1,675.7	2,570.3 (231.7)	487.3 (128.6)	2,083.0
Hospitalized + ICU	224	314	33,938.1 (4,005.9)	23,425.2 (4,276.2)	10,512.9	4,466.0 (677.1)	1,675.6 (688.6)	2,790.4

Diff: mean difference, ICU: intensive care unit

Table J: Mean (SD) SHI-borne direct healthcare and sickness benefit costs in € of incident post-COVID patients and post-COVID-free matched controls over 730-day follow-up, Germany, 2021–2025, sensitivity analyses with newly-occurring diagnosed post-COVID-related health outcomes as censoring event (continued)

	No. of individuals		Outpatient costs			Inpatient costs		
	Post-COVID	Post-COVID-free	Post-COVID	Post-COVID-free	Diff.	Post-COVID	Post-COVID-free	Diff.
**Total population**	18,313	34,838	2,599.5 (26.9)	1,352.8 (32.4)	1,246.6	2,828.5 (77.1)	1,065.7 (71.6)	1,762.8
**Age group**								
0-5 years	50	98	1,011.7 (98.0)	656.3 (60.3)	355.3	502.5 (240.5)	121.0 (92.0)	381.5
6-11 years	177	345	1,188.4 (95.9)	770.9 (127.1)	417.5	1,012.1 (286.8)	396.9 (184.2)	615.2
12-17 years	358	690	1,318.3 (80.5)	725.4 (69.6)	592.9	2,030.9 (626.3)	1,471.2 (619.7)	559.8
18-39 years	4,580	8,806	2,065.0 (40.2)	959.5 (46.4)	1,105.5	1,576.6 (95.8)	759.6 (92.7)	817.0
40-59 years	8,727	16,663	2,547.1 (28.4)	1,326.0 (51.4)	1,221.0	2,447.7 (102.1)	913.0 (67.9)	1,534.7
≥60 years	4,421	8,353	3,432.2 (85.4)	1,931.9 (73.7)	1,500.2	5,051.2 (217.3)	1,708.6 (247.2)	3,342.6
**Prevalent risk condition for severe COVID**								
Yes	13,990	25,091	2,971.7 (33.9)	1,745.2 (48.4)	1,226.5	3,310.2 (95.7)	1,361.7 (102.3)	1,948.5
No	4,323	9,783	1,392.7 (21.3)	612.6 (13.7)	780.0	1,266.8 (99.2)	507.8 (72.7)	759.1
**Severity of first acute diagnosed COVID-19 episode**								
Non-hospitalized	17,050	32,650	2,517.0 (24.8)	1,294.8 (30.0)	1,222.2	2,421.5 (65.5)	820.3 (36.5)	1,601.2
Hospitalized	1,039	1,874	3,733.0 (220.7)	2,279.2 (212.7)	1,453.8	7,535.4 (599.1)	6,077.7 (1,508.7)	1,457.6
Hospitalized + ICU	224	314	4,344.0 (635.2)	4,759.5 (2,209.9)	-415.5	15,577.3 (2,949.1)	7,745.2 (2,901.0)	7,832.0

Diff: mean difference , ICU: intensive care unit

Table J: Mean (SD) SHI-borne direct healthcare and sickness benefit costs in € of incident post-COVID patients and post-COVID-free matched controls over 730-day follow-up, Germany, 2021–2025, sensitivity analyses with newly-occurring diagnosed post-COVID-related health outcomes as censoring event (continued)

	No. of individuals		Prescription drug costs			Prescription medical aid costs		
	Post-COVID	Post-COVID-free	Post-COVID	Post-COVID-free	Diff.	Post-COVID	Post-COVID-free	Diff.
**Total population**	18,313	34,838	2,396.0 (103.5)	1,903.0 (155.9)	493.0	365.0 (11.6)	294.4 (39.3)	70.5
**Age group**								
0-5 years	50	98	236.8 (34.3)	137.2 (29.0)	99.6	30.4 (10.7)	12.2 (7.1)	18.2
6-11 years	177	345	432.9 (107.0)	230.9 (73.2)	202.0	151.9 (72.2)	319.6 (222.9)	-167.7
12-17 years	358	690	1,736.0 (1,220.9)	387.8 (120.6)	1,348.2	117.4 (40.3)	298.4 (147.6)	-181.0
18-39 years	4,580	8,806	1,586.2 (271.8)	747.0 (110.3)	839.3	163.0 (12.1)	135.7 (25.7)	27.2
40-59 years	8,727	16,663	2,116.6 (118.3)	2,255.8 (304.2)	-139.2	336.2 (16.3)	301.1 (78.0)	35.0
≥60 years	4,421	8,353	3,953.1 (206.3)	2,684.1 (198.6)	1,268.9	662.7 (32.7)	448.7 (35.5)	214.0
**Prevalent risk condition for severe COVID**								
Yes	13,990	25,091	2,859.8 (127.0)	2,760.1 (235.4)	99.7	446.8 (15.0)	427.1 (60.1)	19.7
No	4,323	9,783	892.2 (149.8)	285.5 (42.4)	606.7	99.6 (7.2)	44.5 (3.7)	55.1
**Severity of first acute diagnosed COVID-19 episode**								
Non-hospitalized	17,050	32,650	2,223.7 (106.3)	1,620.2 (120.5)	603.5	305.2 (9.9)	255.8 (40.4)	49.4
Hospitalized	1,039	1,874	4,475.4 (385.5)	8,036.3 (2,526.4)	-3,560.9	1,071.0 (106.3)	828.8 (130.2)	242.2
Hospitalized + ICU	224	314	7,386.3 (1,708.4)	6,139.9 (1,466.2)	1,246.4	2,164.4 (361.7)	3,104.9 (872.1)	-940.4
Diff: mean difference, ICU: intensive care unit								

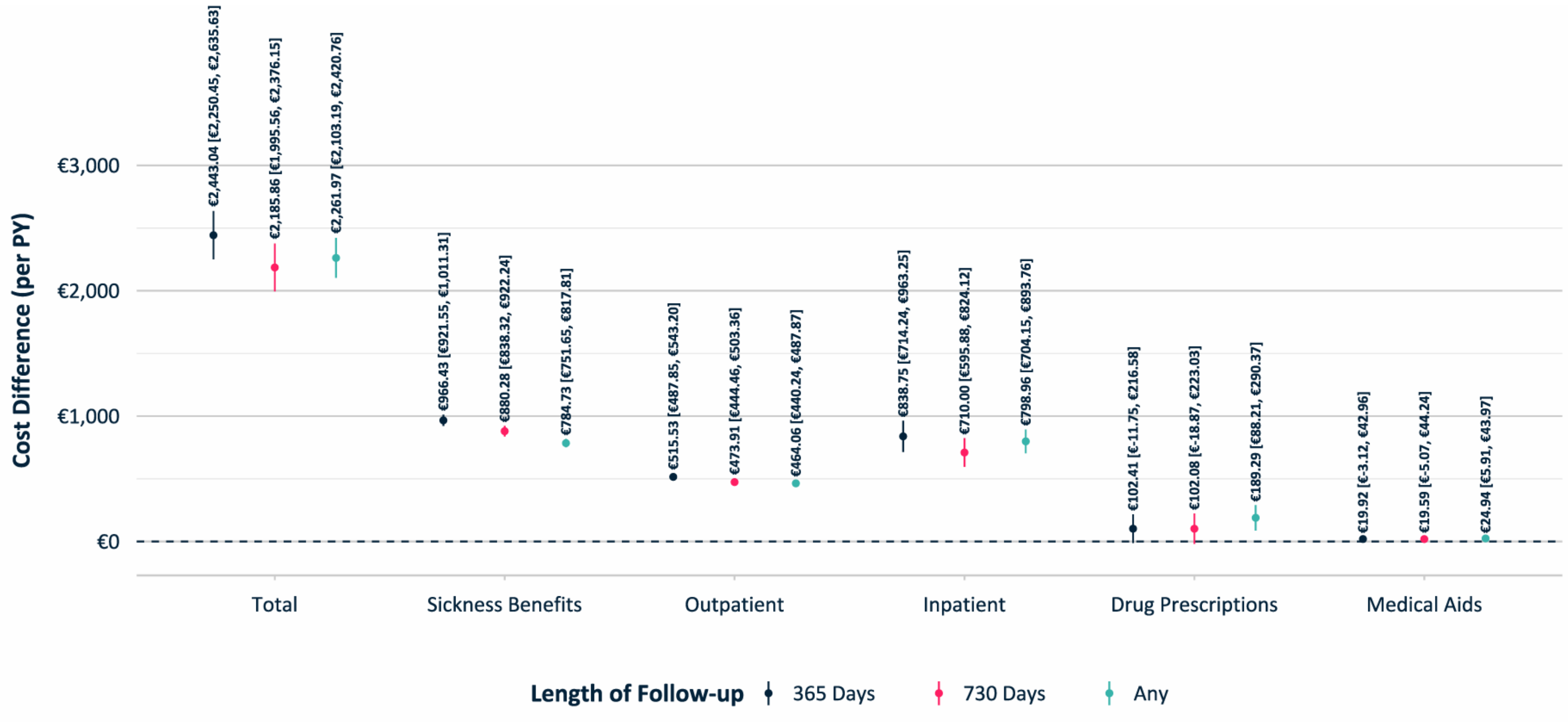

Figure B: Differences in SHI-borne direct healthcare and sickness benefit costs per person-year between post-COVID and post-COVID-free controls over 365 days, 730 days, and any follow-up period, Germany, 2021–2025, sensitivity analyses with newly-occurring diagnosed post-COVID-related health outcomes as censoring events. 95% confidence intervals given in brackets.
